# Cancer-Associated Stromal Fibroblast-Derived Transcriptomes Predict Poor Clinical Outcomes and Immunosuppression in Colon Cancer

**DOI:** 10.3389/pore.2022.1610350

**Published:** 2022-08-04

**Authors:** Jie Wang, Rehana Akter, Md. Fahim Shahriar, Md. Nazim Uddin

**Affiliations:** ^1^ Department of Pharmacy, First Affiliated Hospital of Xinjiang Medical University, Urumqi, China; ^2^ Bioinformatics Research Lab, Center for Research Innovation and Development (CRID), Dhaka, Bangladesh; ^3^ School of Pharmacy, China Pharmaceutical University, Nanjing, China; ^4^ Institute of Food Science and Technology, Bangladesh Council of Scientific and Industrial Research (BCSIR), Dhaka, Bangladesh

**Keywords:** colon cancer, survival time, immunosuppressive roles, metastatic scores, cancer-associated fibroblasts

## Abstract

**Background:** Previous studies revealed that colonic cancer-associated fibroblasts (CAFs) are associated with the modulation of the colon tumor microenvironment (TME). However, identification of key transcriptomes and their correlations with the survival prognosis, immunosuppression, tumor progression, and metastasis in colon cancer remains lacking.

**Methods:** We used the GSE46824, GSE70468, GSE17536, GSE35602, and the cancer genome atlas (TCGA) colon adenocarcinoma (COAD) datasets for this study. We identified the differentially expressed genes (DEGs), Kyoto Encyclopedia of Genes and Genomes (KEGG) pathways, hub genes, and survival-associated genes in colon cancer. Finally, we investigated the correlation of key genes with the survival prognosis, immunosuppression, and metastasis.

**Results:** We identified 246 common DEGs between the GSE46824 and GSE70468 datasets of colonic CAFs, which included 72 upregulated and 174 downregulated genes. The upregulated pathways are mainly involved with cancers and cellular signaling, and downregulated pathways are involved with immune regulation and cellular metabolism. The search tool for the retrieval of interacting genes (STRING)-based analysis identified 15 hub genes and 9 significant clusters in colonic CAFs. The upregulation of *CTHRC1*, *PDGFC*, *PDLIM3*, *NTM*, and *SLC16A3* and downregulation of *FBN2* are correlated with a shorter survival time in colon cancer. The *CTHRC1*, *PDGFC*, *PDLIM3*, and *NTM* genes are positively correlated with the infiltration of tumor-associated macrophages (TAM), macrophages, M2 macrophages, the regulatory T cells (Tregs), T cell exhaustion, and myeloid-derived suppressor cells (MDSCs), indicating the immunosuppressive roles of these transcriptomes in colon cancer. Moreover, the *CTHRC1*, *PDGFC*, *PDLIM3*, *NTM*, and *SLC16A3* genes are gradually increased from normal tissue to the tumor and tumor to the metastatic tumor, and *FBN2* showed the reverse pattern. Furthermore, the *CTHRC1*, *FBN2*, *PDGFC*, *PDLIM3*, and *NTM* genes are positively correlated with the metastatic scores in colon cancer. Then, we revealed that the expression value of *CTHRC1*, *FBN2*, *PDGFC*, *PDLIM3*, *NTM*, and *SLC16A3* showed the diagnostic efficacy in colonic CAFs. Finally, the expression level of *CTHRC1*, *PDGFC*, and *NTM* genes are consistently altered in colon tumor stroma as well as in the higher CAFs-group of TCGA COAD patients.

**Conclusion:** The identified colonic CAFs-derived key genes are positively correlated with survival prognosis, immunosuppression, tumor progression, and metastasis.

## Background

Cancer is a complex ecosystem comprising several cellular components including stromal cancer-associated fibroblasts (CAFs) [1]. CAFs are dominant components of the tumor microenvironment (TME) to control the colon cancer cells [2]. In the TME, CAFs promote the development, invasion, progression, and metastasis of colon cancer cells [2]. The increasing number of CAFs in the TME is correlated with poor prognosis and recurrence of colorectal cancer (CRC) [3]. Colonic CAFs-derived transcriptomes are associated with tumor angiogenesis, metastasis, and prognosis of human colon cancer [4]. In adenomatous colorectal polyps and primary tumors, accumulation of CAFs is associated with inferior prognosis and recurrence of disease [5]. CAFs of colonic mucosa provide information on functional heterogeneity and are associated with the prognosis [6]. The CAFs-associated markers are increased at the invasive front of the tumor and it is critically correlated with the tumor-stroma ratio in CRC [7].

CAFs are predominantly associated with the secretion of several inflammatory mediators which included chemokines, cytokines, transcription factors, proteases, and growth factors, thereby promoting the immunosuppressive TME [8]. These immunosuppressive mediators strictly control the reactivity of tumor and stroma cell communication in the TME [9, 10]. CAFs are associated with the modulation of the immune system and it is correlated with immune evasion and poor immunotherapy responses [11]. Moreover, CAFs- derived gene expression signatures are associated with poor clinical outcomes in CRC [12, 13]. Hang Zheng et al. identified the CAFs-associated transcriptomes that influence the prognosis and therapeutic responses in CRC [14]. In CRC, the higher CAF infiltration scores predicted poor disease-free survival (DFS) outcomes [15]. CAFs- expressed transcription factors (TFs) are correlated with epithelial-to-mesenchymal transition (EMT) which ultimately controls the cellular stemness and fate [16]. Also, TFs are related to the infiltration of CAFs and the independent prognostic factors which ultimately associated with cancer cell invasion, proliferation, and progression [17]. These studies provide the clue that the CAFs-derived transcriptomes are associated with cancer initiation, invasion, migration, and metastasis.

Herein, we identified the deregulated transcriptional signatures of human colonic CAFs. Then we analyzed the association of CAFs-derived DEGs with the enrichment of pathways. Moreover, we identified the key hub genes and significant clusters from the protein-protein interaction (PPI) network. Furthermore, we investigated the correlation of key genes with survival prognosis, immunosuppression, tumor progression, and metastasis.

## Methods

### Datasets

We searched the NCBI gene expression omnibus (GEO) database (https://www.ncbi.nlm.nih.gov/geo/) using the keywords “colon cancer fibroblast,” “cancer-associated fibroblast,” “CAFs” “stroma cells,” “colon tumor stroma cells” and “tumor stroma,” and identified two cancer-associated fibroblasts gene expression datasets GSE46824 (*n* = 34) [18] and GSE70468 (*n* = 14) [19] of colonic CAFs. In GSE70468, we selected the 7 CAFs samples and 7 colon normal fibroblasts samples and removed the other samples from this dataset. Besides, we downloaded the TCGA COAD cohort and normalized the data into log_2_-base transformation (https://gdc-portal.nci.nih.gov/) [20]. For analyzing the survival differences in the TCGA COAD dataset (https://gdc-portal.nci.nih.gov/), we used the gene expression profiling interactive analysis (GEPIA) [21] tool (http://gepia2.cancer-pku.cn/#index). Furthermore, we used PrognoScan-based [22] colon cancer data, the GSE17536 (*n* = 177) [23], for validating the survival-associated genes found in TCGA data. Finally, we used the colon tumor-stromal dataset GSE35602 [24] for verifying the expression levels of key genes. The summary of the GEO datasets is presented in Table 1.

**TABLE 1:** The summary of the GEO datasets that are used in this study.

Serial number	Accession number	Title	Platform	Samples number
1.	GSE46824	Carcinoma-associated fibroblasts’ transcriptomic program predicts clinical outcome in stage II/III colorectal cancer	GPL6244	*n* = 34
2.	GSE70468	Vitamin D receptor expression and associated gene signature in tumor stromal fibroblasts predict clinical outcome in colorectal cancer	GPL17077	*n* = 14
3.	GSE35602	microRNA and Gene expression profiles in colorectal cancer stromal tissue	GPL6480	*n* = 17
4.	GSE17536	Metastasis Gene Expression Profile Predicts Recurrence and Death in Colon Cancer Patients	GPL570	(*n* = 177)

### Identification of Differentially Expressed Genes

The differential expression was screened by using GEO2R (http://www.ncbi.nlm.nih.gov/geo/geo2r), which is an interactive web tool to identify the DEGs. GEO2R tool identified the significant DEGs by utilizing the GEOquery [25] and limma R packages [26] from the Bioconductor project (http://www.bioconductor.org/). The R package “limma” was employed for identifying the significant DEGs between CAFs and normal colonic fibroblasts (NCFs) [26]. We identified the DEGs with a threshold absolute value of fold change (LogFC) > 0.585 and a *p*-value ≤0.05 [27, 28]. Finally, we identified the common DEGs between both datasets. We utilized the online tool “Calculate and draw custom Venn diagrams” (http://bioinformatics.psb.ugent.be/webtools/Venn/) for identifying common DEGs in both datasets.

### Gene-Set Enrichment Analysis

We performed a gene-set enrichment analysis of the DEGs by using the gene-set enrichment analysis (GSEA) [29]. We inputted all significant commonly found upregulated and downregulated DEGs into the GSEA tool for identifying deregulated pathways. In the GSEA, we selected the KEGG pathways panel to identify the significant pathways. The KEGG [30] pathways significantly associated with the upregulated DEGs and the downregulated DEGs were identified, respectively. The *p*-value <0.05 was considered significant when separating the pathways.

### Identifying Hub Genes and Modular Analysis From Protein-Protein Interaction Network of Differentially Expressed Genes

To better know the relationship among these identified DEGs, the PPI network was established using the STRING-based analysis [31]. To identify the rank of hub genes, we used Cytoscape plug-in tool cytoHubba [32]. Hub genes were identified based on the degree of interactions with neighbour genes. We set the minimum required interaction score as 0.40 for identifying the PPI of DEGs. Hub genes were defined as a gene that was connected to a minimum of 10 other DEGs in the PPI. We visualize the PPI networks by utilizing the Cytoscape 3.6.1 software [33]. A Cytoscape plug-in molecular complex detection (MCODE) was employed to detect the modules from the PPI network [34]. This is a computational method for searching molecular complexes in large protein interaction networks. We identified the significant modules based on the MCODE score and node number. The threshold of the MCODE was node score cut-off: 0.2, haircut: true, k-core: 2, and maximum depth from Seed: 100.

### Survival Analysis of Differentially Expressed Genes by Using the Gene Expression Profiling Interactive Analysis and PrognoScan

We compared the overall survival (OS) and the DFS of colon cancer patients. Kaplan-Meier survival curves were used to show the survival differences between the high expression group and low expression groups. The survival significance of all DEGs in the TCGA COAD cohort was analysed using GEPIA [21] databases. GEPIA is a web-based tool to deliver fast and customizable functionalities based on TCGA data. Furthermore, we used PrognoScan-based [22] GSE17536 (*n* = 177) [23] for validating the survival-associated genes found in the TCGA COAD dataset. PrognoScan is a database for meta-analysis of the prognostic value of genes. Cox regression *p*-value <0.05 was considered as significant when comparing the survival between the two groups.

### The ESTIMATE Algorithm for Quantifying Immune Score and Stromal Score

ESTIMATE is an algorithmic tool based on the R package for predicting tumor purity, immune score (predicting the infiltrations of immune cells), and stromal score (predicting the infiltrations of stromal cells) which uses the gene expression profiles of 141 immune genes and 141 stromal genes [35]. The presence of infiltrated immune cells and stromal cells in tumor tissues were calculated using related gene expression matrix data, represented by immune score and stromal score, respectively [35]. Then we calculated the correlations of key genes with immune scores and stromal scores. The threshold value of correlation is R > 0.30, and *p*-value is not less than 0.001 (Spearman’s correlation test).

### Single-Sample Gene-Set Enrichment Analysis and Correlation of Immune Signatures With Survival-Associated Genes

One of the extension packages of GSEA, single-sample gene-set enrichment analysis (ssGSEA) was used to identify the enrichment scores of immune cells for each pairing of a sample and gene set in the tumor samples [36]. We collected the marker gene set for immune signatures and utilized each gene set to quantify the ssGSEA scores of specific immune signatures [37–40]. We identified the ssGSEA scores of CD8^+^ T cells, CD4^+^ regulatory T cells, NK cells, TAM, macrophages, M2 macrophages, Tregs, T cell exhaustion, myeloid-derived suppressor cells (MDSCs), and CAFs. Moreover, we used the metastasis-promoting gene set for identifying the ssGSEA scores of metastasis-promoting genes [40, 41]. All of the marker genes are displayed in Supplementary Table S1. Then we investigated Spearman’s correlation between the ssGSEA scores and specific survival-associated genes. The absolute value of this correlation was greater than 0.30 with a *p*-value less than 0.001.

### Diagnostic Efficacy Evaluation and Expression Validation for Survival-Associated Key Genes

To assess diagnostic values of the survival-associated genes, the receiver operating characteristic (ROC) curve was plotted and the area under the ROC curve (AUC) was calculated using the “pROC” R package [42] to evaluate the capability of distinguishing cancer-associated fibroblasts and normal tissues. The “pROC” R package can visualize, smooth, and compare ROC curves. The AUC can be compared with statistical tests based on U-statistics or bootstrap. A greater AUC value of individual genes indicated the differences between tumor and normal samples, and the key gene of AUC > 0.5 in the CAFs datasets was defined as a diagnostic efficiency of the gene [43]. To verify the survival-associated genes in a colorectal cancer stromal tissue, we selected GSE35602 [24] to distinguish the expression levels of key genes between non-tumor and tumor samples. We identified the ssGSEA scores (prediction of the content) of CAFs by using the marker genes (Supplementary Table S1) of CAFs. Then, we divided the TCGA COAD samples into the high-CAFs group versus the low-CAFs group based on the median value of ssGSEA scores of CAFs.

### Statistical Analysis

We used the R software version 4.0.1 for all statistical analysis. In the Log-rank test, *p* < 0.05 was considered statistically significant for survival analysis. To investigate the correlation of survival-associated genes, Spearman’s correlation between the ssGSEA scores and specific survival-associated genes was performed (*p* < 0.001). We used Welch’s t-test for identifying the significance of specific genes between the two groups of samples (*p* ≤ 0.05). We utilized the R package ggplot2 for the graphical presentation of the Heatmap and correlation graph.

## Results

### Identification of Differentially Expressed Genes in Colonic Cancer-Associated Fibroblasts

**FIGURE 1:**
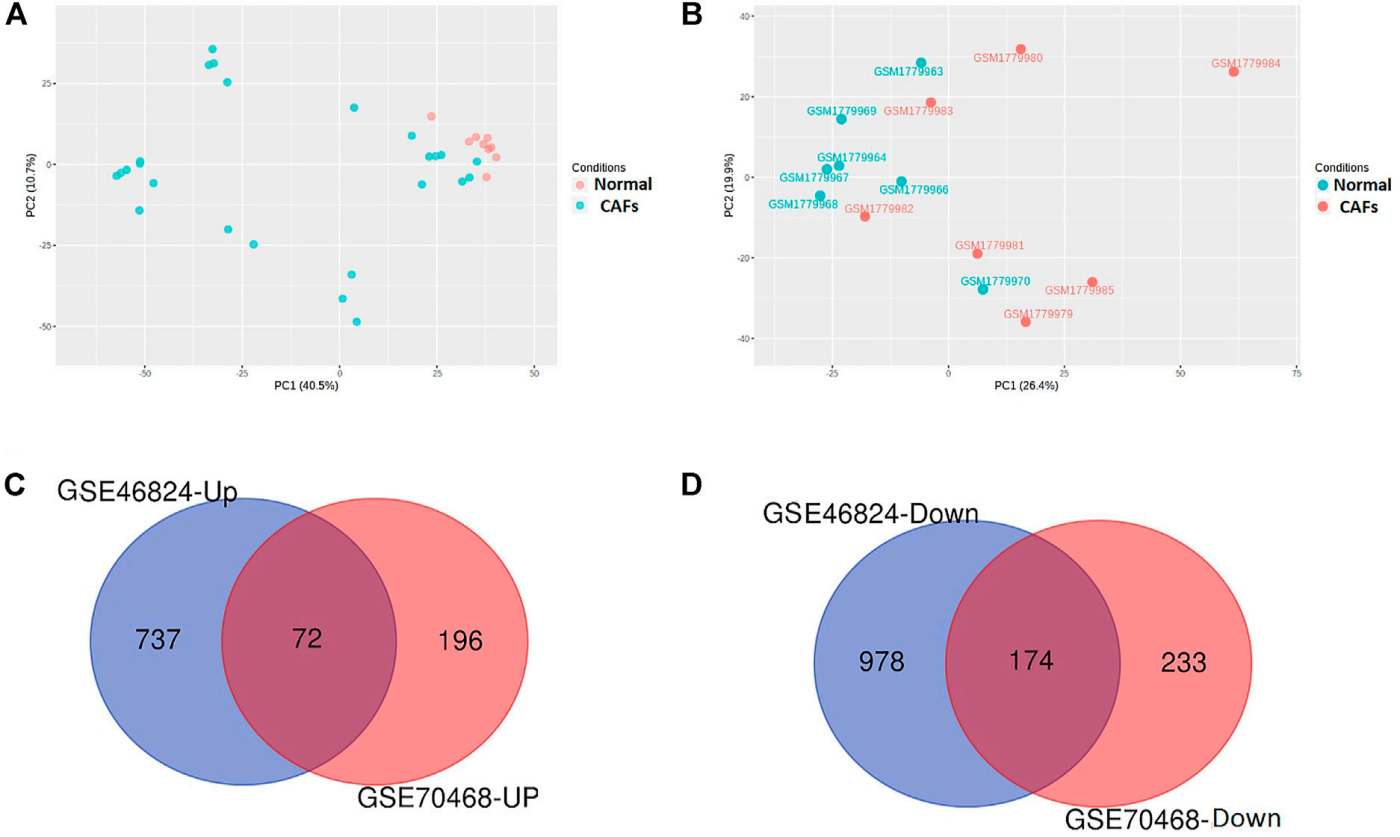
Identification of DEGs in the colonic CAFs. **(A)** The PCA analysis of samples in the GSE46824 dataset comprehensively summarizes the data **(B)**. The PCA analysis of samples in the GSE70468 dataset comprehensively summarizes the data. **(C)** Identifying commonly found 72 upregulated genes in both datasets. **(D)** Identifying commonly found 174 downregulated genes in both datasets. CAFs: cancer-associated colonic fibroblasts; Normal: normal colonic fibroblasts; GSE46824-Up: Upregulated gene in GSE46824; GSE46824-Down: Downregulated gene in GSE46824; GSE70468-Up: Upregulated gene in GSE70468, GSE70468-Down: Downregulated gene in GSE70468.

We identified 246 commonly differentially expressed genes (DEGs) between the GSE46827 and GSE70468 datasets. The principal component analysis (PCA) plot of samples is shown in Figures 1A,B and the commonly upregulated and downregulated genes in CAFs and normal colonic fibroblasts (NCFs) are shown in Figures 1C,D respectively. We found 72 upregulated (Figure 1C; Supplementary Table S2) and 174 downregulated (Figure 1D; Supplementary Table S3) genes in the CAFs when compared with NCFs. *CDH13*, *DSP*, *EFNB2*, *IL7R*, *KIAA1217*, *KRT19*, *NTM*, *PDLIM3*, *SRGN*, *SYNPO2L*, *TGFB2*, and *TNFSF4* were the most significantly upregulated genes in colon CAFs (Supplementary Table S2), while *A2M*, *AOC3*, *ADH1B*, *ASPA*, *CHRDL1*, *COL14A1*, *CYP24A1*, *FBN2*, *FBLN1*, *GREM2*, *IL33*, *NOVA1*, *PRKCH*, *SEPP1*, and *SMOC2* are the most downregulated genes over the selected datasets (Supplementary Table S3).

### CAFs-Derived Transcriptomes Are Associated With the Enrichment of Kyoto Encyclopedia of Genes and Genomes Pathways

**FIGURE 2:**
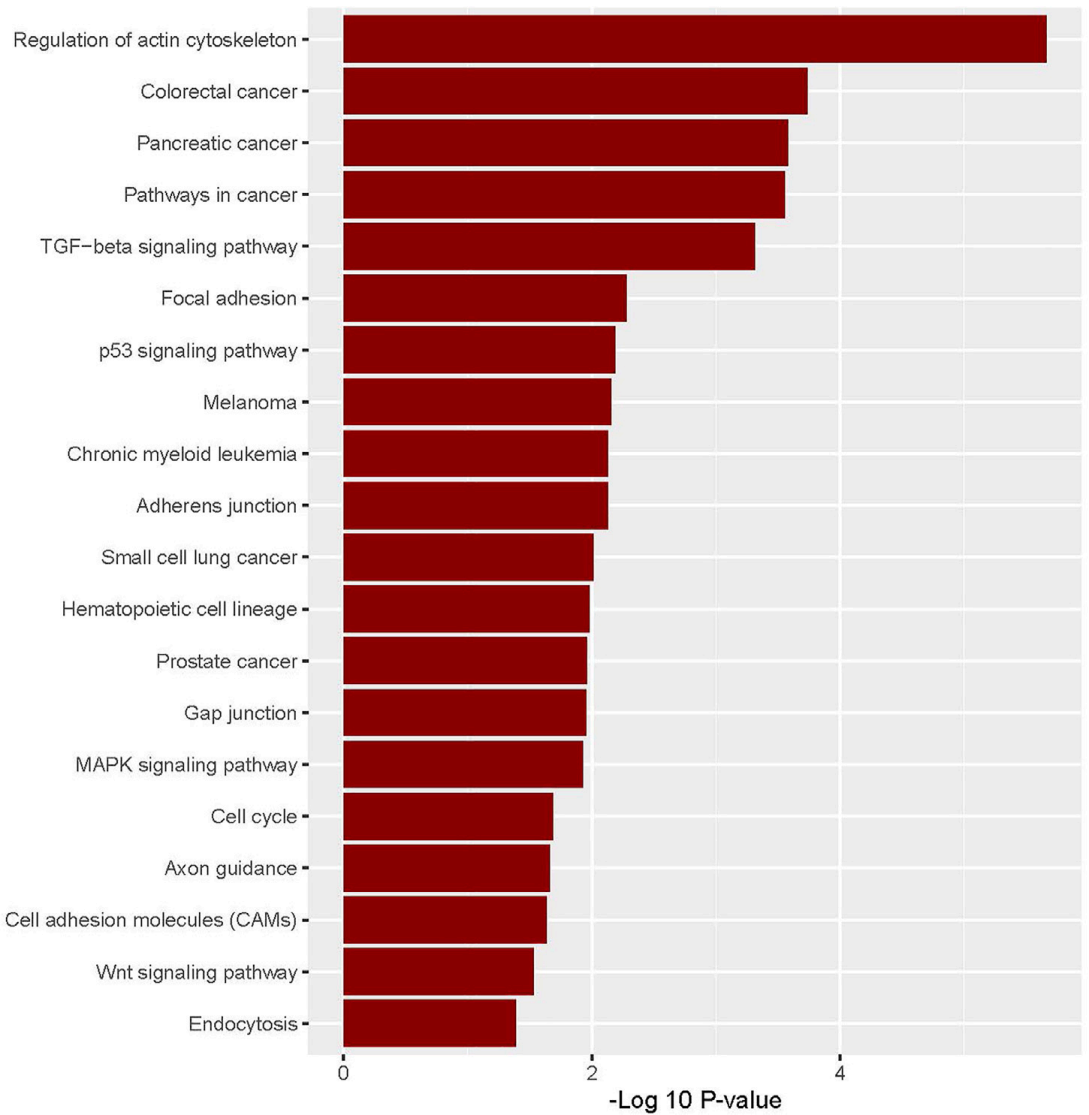
Significantly enriched KEGG pathways that are associated with upregulated colonic CAFs-derived DEGs.

**FIGURE 3:**
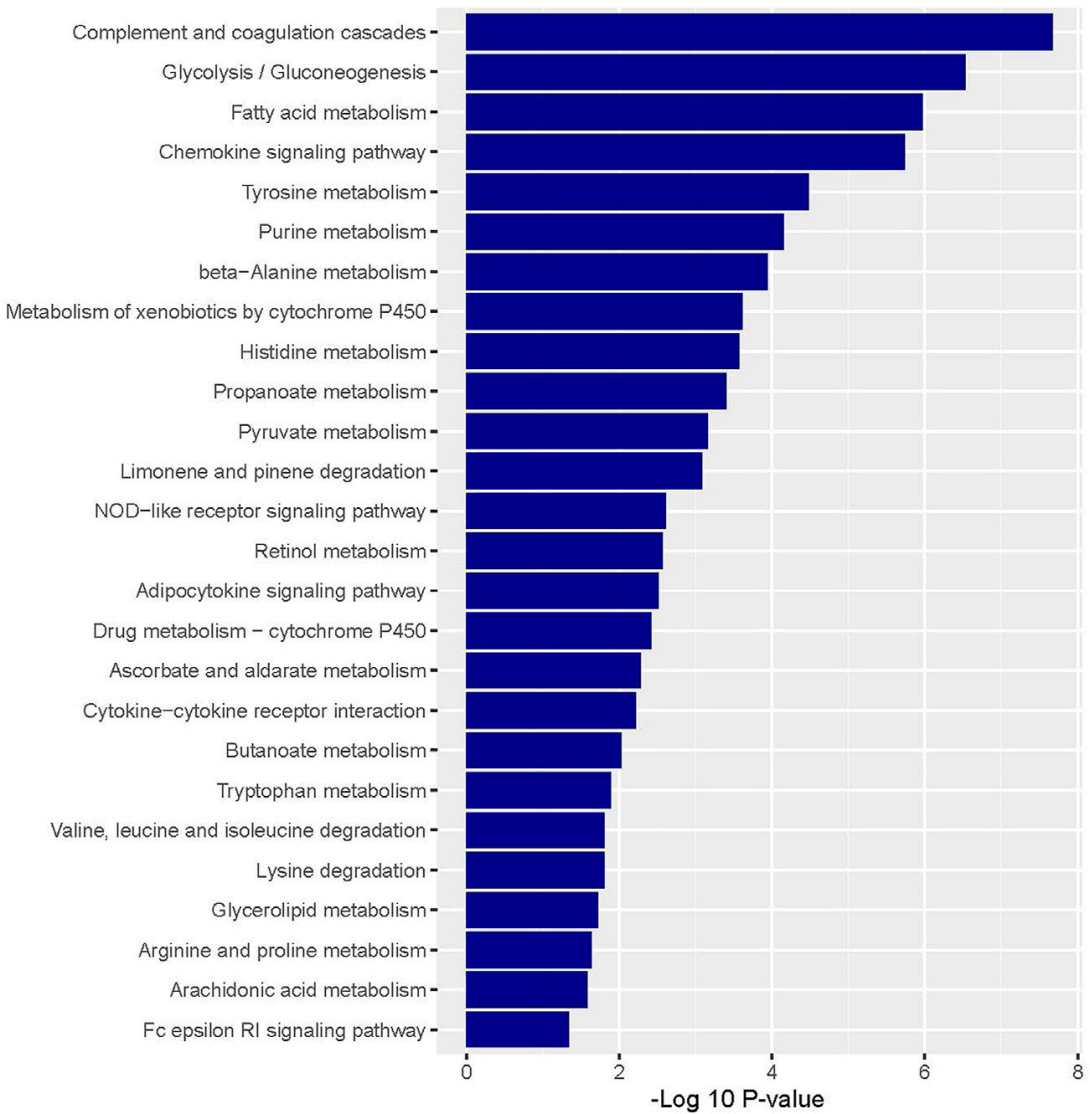
Significantly enriched KEGG pathways that are associated with downregulated colonic CAFs-derived DEGs.

The enriched upregulated and downregulated biological pathways were identified by using the GSEA tool (Figures 2, 3). GSEA identified 28 KEGG pathways significantly associated with common upregulated 72 DEGs (Supplementary Table S4). The pathways are mainly involved with cancer or malignant tumor (colorectal cancer, pancreatic cancer, pathways in cancer, melanoma, chronic myeloid leukemia, small cell lung cancer, and prostate cancer) and cellular signaling and developments (regulation of actin cytoskeleton, TGF-beta signaling pathway, focal adhesion, p53 signaling pathway, adherens junction, gap junction, MAPK signaling pathway, cell cycle, cell adhesion molecules (CAMs), and Wnt signaling pathway) (Figure 2).

Besides, we identified the 54 KEGG pathways that are significantly linked with commonly found downregulated 174 DEGs (Supplementary Table S5). The pathways are involved with immune regulation (such as complement and coagulation cascades, chemokine signaling pathway, and cytokine-cytokine receptor interaction) and cellular metabolism (glycolysis/gluconeogenesis, fatty acid metabolism, tyrosine metabolism, purine metabolism, beta-Alanine metabolism, metabolism of xenobiotics by cytochrome P450, histidine metabolism, propanoate metabolism, pyruvate metabolism, limonene and pinene degradation, retinol metabolism, drug metabolism—cytochrome P450, ascorbate and aldarate metabolism, butanoate metabolism, tryptophan metabolism, lysine degradation, valine, leucine and isoleucine degradation, glycerolipid metabolism, arginine and proline metabolism, and arachidonic acid metabolism) (Figure 3).

### STRING-Based Protein-Protein Interaction Analysis Identified CAFs-Derived Hub Genes and Significant Modules in Colon Cancer

We investigated the PPI of all significant CAFs-derived DEGs. The PPI information of STRING is inputted into the Cytoscape for identifying and visualizing the hub genes and significant clusters. We identified 15 hub genes (minimum degree of interaction is 10 with other DEGs) which included 3 upregulated hub genes (*PLAUR*, *RAC2*, and *TGFB2*) and 12 downregulated hub genes (*A2M*, *ADCY3*, *ADCY4*, *ADCY9*, *BIN1*, *CFD*, *CLU*, *CXCL12*, *LDLR*, *PPARG*, *PTK2B*, and *VTN*) (Table 2).

**TABLE 2:** Identification of the 15 hub genes and their degree of interaction with differential expression between the CAFs and NCFs in two datasets.

Rank	Name of genes	Degree of interaction	Regulatory status	GSE46824		GSE70468	
				LogFC	*p*-value	LogFC	*p*-value
1	*CLU*	18	Downregulated	−1.01	1.09E-04	−1.14	4.56E-03
1	*CXCL12*	18	Downregulated	−1.42	4.25E-03	−1.58	1.82E-02
3	*VTN*	15	Downregulated	−1.45	1.78E-04	−1.09	3.18E-03
4	*ADCY3*	13	Downregulated	−0.78	3.35E-05	−0.76	3.98E-03
5	*A2M*	12	Downregulated	−1.94	2.15E-03	−2.13	8.36E-03
5	*LDLR*	12	Downregulated	−0.67	9.72E-03	−0.65	3.43E-02
5	*TGFB2*	12	Upregulated	2.31	3.57E-09	0.59	1.35E-02
5	*PPARG*	12	Downregulated	−1.60	1.18E-05	−0.86	1.21E-02
5	*ADCY9*	12	Downregulated	−0.73	2.83E-03	−0.98	4.91E-04
5	*PLAUR*	12	Upregulated	0.68	3.10E-03	0.98	1.28E-02
11	*ADCY4*	11	Downregulated	−0.67	7.70E-04	−1.21	2.99E-03
11	*PTK2B*	11	Downregulated	−0.74	1.73E-04	−0.66	1.20E-02
13	*RAC2*	10	Upregulated	1.02	8.65E-05	0.81	4.43E-02
13	*CFD*	10	Downregulated	−2.40	6.34E-08	-3.60	1.20E-03
13	*BIN1*	10	Downregulated	−0.73	1.34E-06	-0.83	1.35E-02

We found that 183 DEGs were involved in PPI (Supplementary Table S6). The other DEGs were not involved in the PPI network. We investigated the significant cluster-based analysis of this PPI network. The MCODE-based analysis identified 9 clusters from the original PPI networks. The description of MCODE-derived clusters is illustrated in Table 3. The top significant cluster 1 included 7 nodes and 21 edges (Table 3). We identified the significantly enriched KEGG pathways for the identified 9 clusters by using the GSEA. Interestingly, we found that seven clusters are associated with the enrichment of KEGG pathways (*p* < 0.05). Gene set of cluster 1 is associated with the enrichment of complement and coagulation cascades (*p* = 1.67e-7). Gene set of cluster 2 is mainly involved with immune regulation and cellular signaling (Table 3). In addition, the gene set of cluster 3 is mainly associated with the regulation of metabolism (Table 3). Also, Cluster 4, Cluster 6, Cluster 8, and Cluster 9 are associated with the enrichment of KEGG pathways (Table 3).

**TABLE 3:** MCODE identified significant 9 clusters from the PPI networks of DEGs and the GSEA-based analysis identified the enriched KEGG pathways (*p* < 0.05) for a specific gene set of individual clusters. NA: KEGG pathways not found for a specific cluster.

Cluster	Score of MCODE	Nodes	Edges	Node symbol	Enrichment of KEGG pathways (*p* **<** 0.05)
1	7	7	21	*PROS1*, *ISLR*, *TGFB2*, *CFD*, *SRGN*, *A2M*, *CLU*	Complement and coagulation cascades
2	6.75	9	27	*ADCY9*, *ADORA1*, *ADCY4*, *PTGER3*, *CCL13*, *CXCL12*, *ADCY3*, *GCH1*, *PDE3B*	Chemokine signaling pathway, progesterone-mediated oocyte maturation, purine metabolism, calcium signaling pathway, dilated cardiomyopathy, gap junction, GnRH signaling pathway, melanogenesis, Oocyte meiosis, vascular smooth muscle contraction, vasopressin-regulated water reabsorption, vibrio cholerae infection, cytokine-cytokine receptor interaction, and neuroactive ligand-receptor interaction
3	5	5	10	*ALDH2*, *ADH1A*, *ALDH3A2*, *ADH1B*, *ADH1C*	Fatty acid metabolism, glycolysis/gluconeogenesis, tyrosine metabolism, retinol metabolism, metabolism of xenobiotics by cytochrome P450, drug metabolism-cytochrome P450, limonene and pinene degradation, beta-Alanine metabolism, ascorbate and aldarate metabolism, histidine metabolism, propanoate metabolism, butanoate metabolism, pyruvate metabolism, tryptophan metabolism, lysine degradation, valine, leucine and isoleucine degradation, glycerolipid metabolism, and arginine and proline metabolism
4	4	8	14	*VTN*, *PKP2*, *DSG2*, *C1S*, *DSP*, *C1R*, *PPL*, *KRT8*	Arrhythmogenic right ventricular cardiomyopathy (ARVC), complement and coagulation cascades, and systemic lupus erythematosus
5	4	4	6	*ATP8B4*, *TNFRSF1B*, *PLAUR*, *PGRMC1*	NA
6	3.333	4	5	*SEMA3B*, *NTF3*, *EFNB2*, *SLIT3*	Axon guidance
7	3	3	3	*RSPO3*, *FZD1*, *RSPO2*	NA
8	3	3	3	*F2R*, *GNA14*, *F2RL2*	Calcium signaling pathway and Neuroactive ligand-receptor interaction
9	3	3	3	*NFASC*, *CADM1*, *EPB41L3*	Cell adhesion molecules (CAMs)

### Cancer-Associated Fibroblasts Derived Transcriptomes Are Correlated With Survival Prognosis in Colon Cancer

**FIGURE 4:**
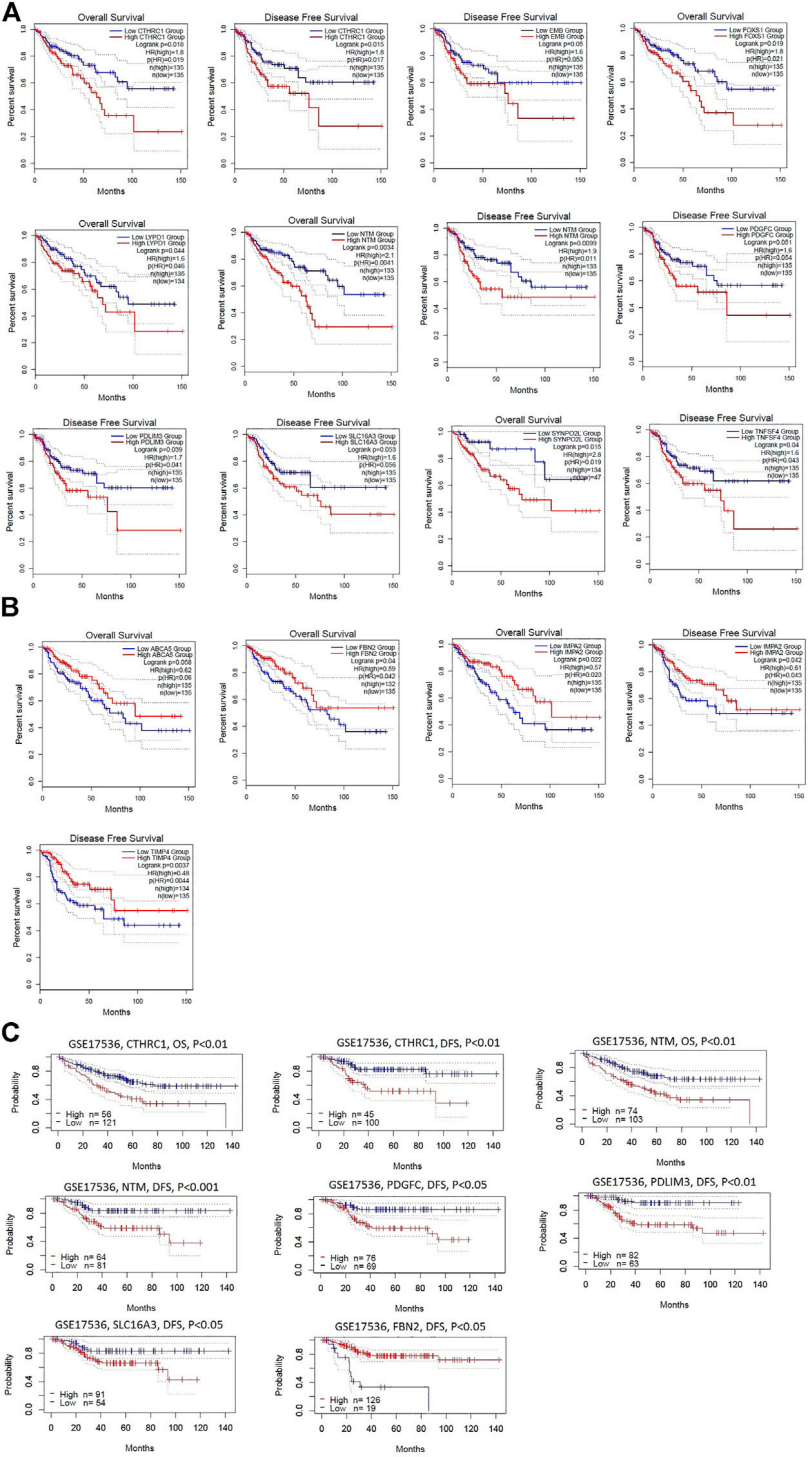
Identification of survival-associated DEGs in colonic CAFs. **(A)** The higher expression group of upregulated genes that included *CTHRC1*, *EMB*, *FOXS1*, *LYPD1*, *NTM*, *PDGFC*, *PDLIM3*, *SLC16A3*, *SYNPO2L*, and *TNFSF4* are significantly correlated with shorter survival time in the TCGA COAD cohort. **(B)** The lower expression group of downregulated genes that included *ABCA5*, *FBN2*, *IMPA2*, and *TIMP4* are significantly correlated with shorter survival time in the TCGA COAD cohort **(C)**. The higher group of *CTHRC1*, *NTM*, *PDGFC*, *PDLIM3*, and *SLC16A3* genes are consistently correlated with the shorter survival of colon cancer patients in GSE17536. In contrast, the lower group of *FBN2* gene is consistently correlated with the shorter survival of colon cancer patients in GSE17536.

We investigated the survival significance of the CAFs-derived all significant common DEGs (72 upregulated and 174 downregulated genes) in TCGA COAD data. Our analysis revealed that the upregulated group of CAFs-derived upregulated genes included *CTHRC1*, *EMB*, *FOXS1*, *LYPD1*, *NTM*, *PDGFC*, *PDLIM3*, *SLC16A3*, *SYNPO2L*, and *TNFSF4* (Figure 4A) and the lower expression group of downregulated genes included *ABCA5*, *FBN2*, *IMPA2*, and *TIMP4* (Figure 4B) are significantly correlated with shorter survival time of colon cancer patients (Figure 4). Moreover, we verified this result in a GEO dataset GSE17536 (n = 177) of colon cancer. We found that upregulated group of *CTHRC1*, *NTM*, *PDGFC*, *PDLIM3*, and *SLC16A3* genes are consistently correlated with the shorter survival of colon cancer patients in GSE17536 (Figure 4C). In contrast, the downregulated group of *FBN2* gene is consistently correlated with the shorter survival of colon cancer patients in GSE17536 (Figure 4C), indicating the tumor-suppressive role of *FBN2* in colon cancer.

### Association of Survival-Associated Genes With Immune Score and Stroma Score in Colon Cancer

**FIGURE 5:**
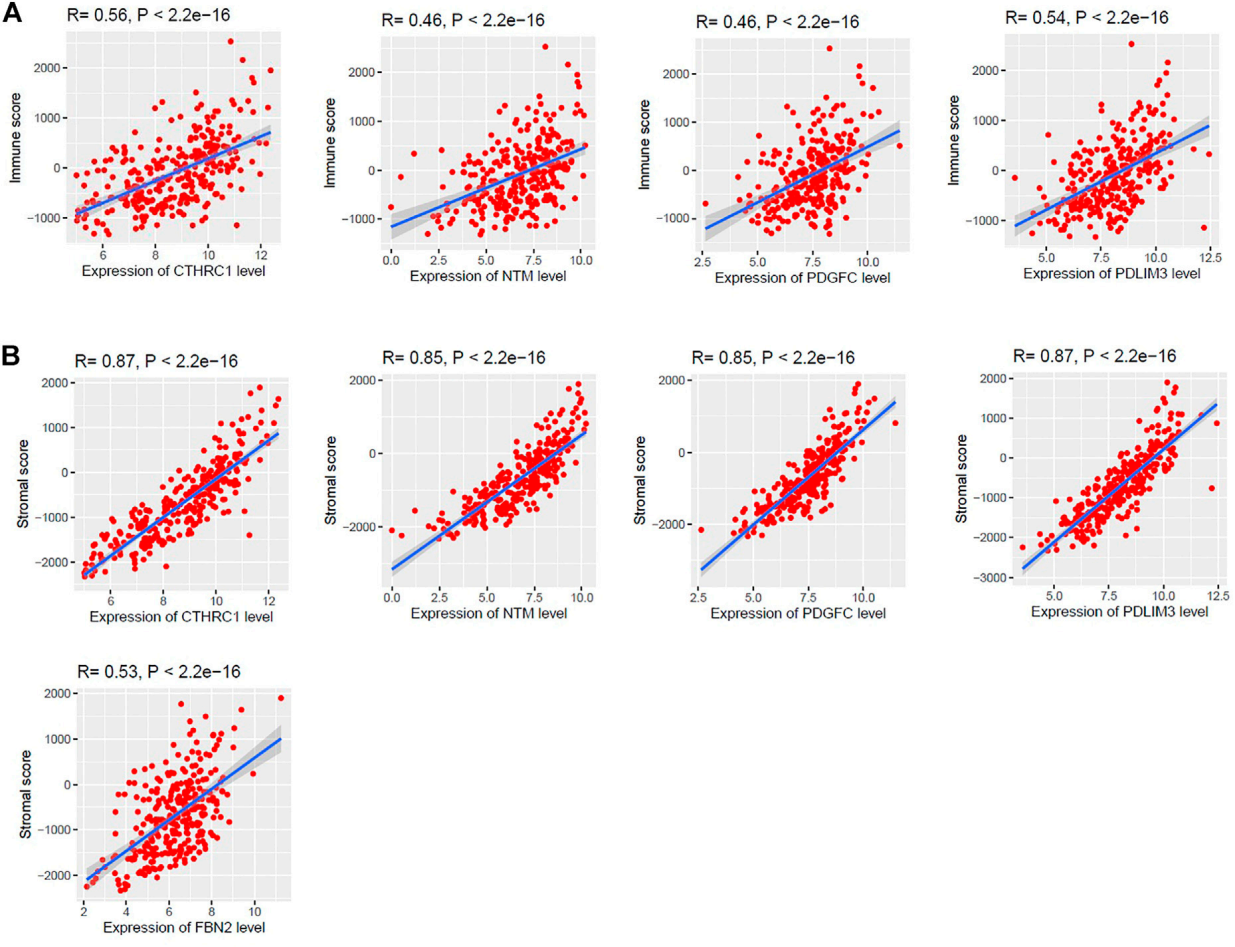
Association of *CTHRC1*, *NTM*, *PDGFC*, *PDLIM3*, and *FBN2* expression levels with the regulation of tumor microenvironment (TME) in colon cancer. **(A)** The expression levels (log2 transformation) of *CTHRC1*, *NTM*, *PDGFC*, and *PDLIM3* are moderately correlated with the immune score. **(B)** Strong positive correlation between *CTHRC1*, *NTM*, *PDGFC*, *PDLIM3*, and *FBN2* with stromal scores. (R is Spearman’s correlation coefficient and P is the *p*-value). We used the TCGA COAD cohort (*n* = 287) to identify the correlation.

Since the upregulated group of *CTHRC1*, *NTM*, *PDGFC*, *PDLIM3*, and *SLC16A3* and the downregulated group of *FBN2* genes are consistently correlated with the shorter survival time in TCGA and GEO datasets, we investigated the association of these genes with immune score and stromal scores in colon cancer. Interestingly, we found that the expression levels (log2 transformation) of *CTHRC1*, *NTM*, *PDGFC*, and *PDLIM3* are moderately correlated with immune scores (Spearman’s correlation test, *p* < 0.001) (Figure 5A). In addition, the expression levels (log2 transformation) of *CTHRC1*, *NTM*, *PDGFC*, *PDLIM3*, and *FBN2* are strongly correlated with the stromal score (Spearman’s correlation test, *p* < 0.001) (Figure 5B). In contrast, *SLC16A3* is not correlated with immune scores and stromal scores in the TCGA-COAD cohort (R > 0.30 and *p* < 0.01). This indicated that the expression levels of *CTHRC1*, *FBN2*, *NTM*, *PDGFC*, and *PDLIM3* genes are associated with the modulation of immune and stromal activity in the colon cancer tumor microenvironment.

### Cancer-Associated Fibroblasts Derived Survival-Associated Genes Are Associated With Immunosuppression in Colon Cancer

**FIGURE 6:**
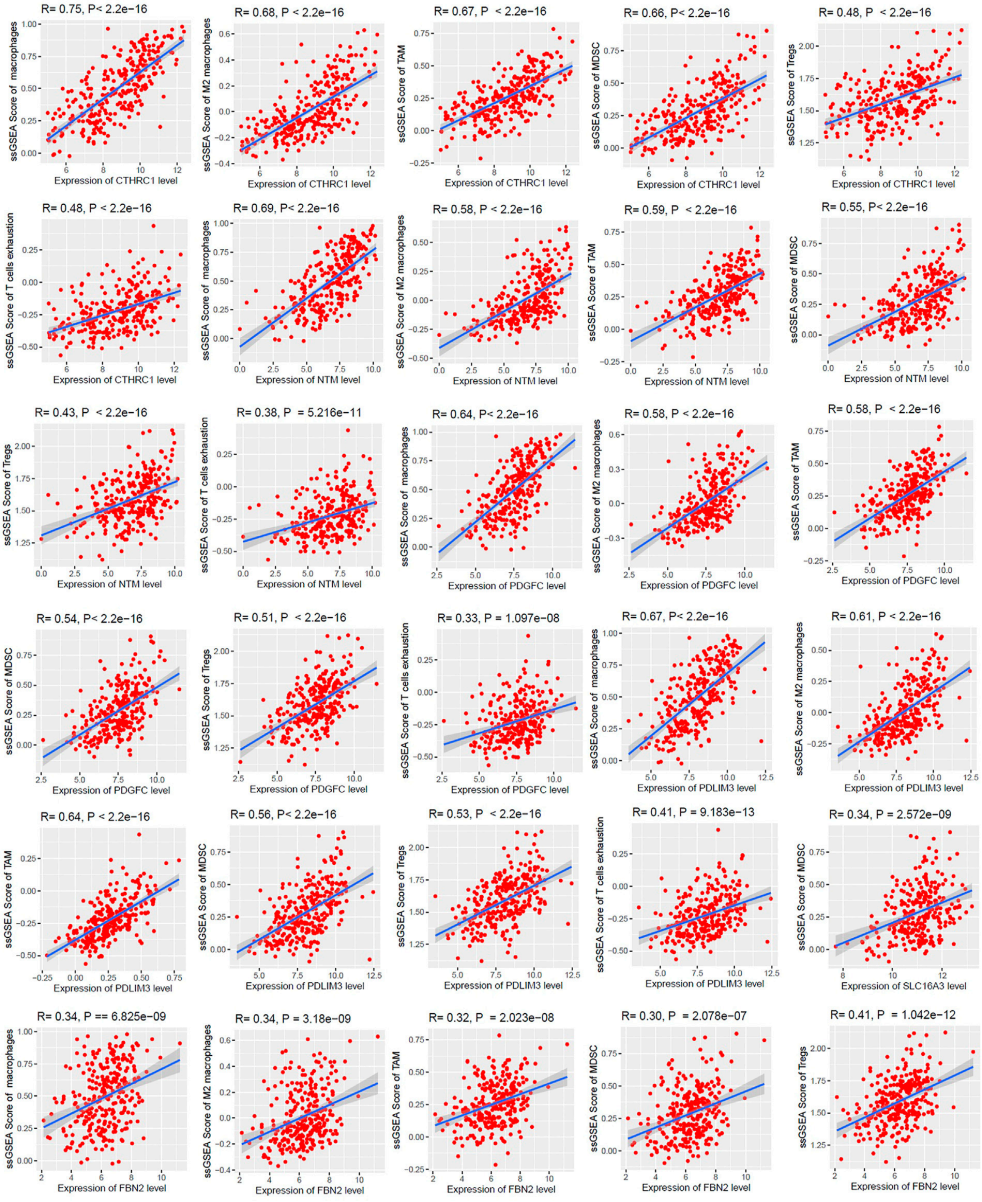
The expression levels of *CTHRC1*, *NTM*, *PDGFC*, *PDLIM3*, *SLC16A3*, and *FBN2* are positively correlated with ssGSEA scores of immune signatures in colon cancer (Spearman’s correlation test, *p* < 0.001). We used the TCGA COAD cohort (*n* = 287) to identify the correlations.

Since survival time of patients is correlated with immunological responses in human cancers [44], we investigated the correlation of six survival-associated genes (*CTHRC1*, *NTM*, *PDGFC*, *PDLIM3*, *SLC16A3*, and *FBN2*) with the several immune stimulatory and inhibitory signatures including CD8^+^ T cells, CD4^+^ regulatory T cells, NK cells, TAM, macrophages, M2 macrophages, Tregs, T cell exhaustion, and MDSCs. We found that the expression levels of upregulated *CTHRC1*, *NTM*, *PDGFC*, and *PDLIM3* are positively correlated with ssGSEA scores of TAMs, macrophages, M2 macrophages, Tregs, T cell exhaustion, and MDSCs (Spearman’s correlation test, *p* < 0.001) (Figure 6). Besides, *SLC16A3* is positively correlated with the infiltration of MDSCs (Spearman’s correlation test, *p* < 0.001) (Figure 6). In addition, the expression level of *FBN2* is correlated with ssGSEA scores of TAMs, macrophages, M2 macrophages, Tregs, and MDSCs (Spearman’s correlation test, *p* < 0.001). Interestingly, the CD8^+^ T cells, CD4^+^ regulatory T cells, and NK cells are not significantly correlated with the expression levels of *CTHRC1*, *NTM*, *PDGFC*, *PDLIM3*, *SLC16A3*, and *FBN2.*

### Cancer-Associated Fibroblasts Derived Survival-Associated Genes Are Associated With Tumor Progression and Metastasis in Colon Cancer

**FIGURE 7:**
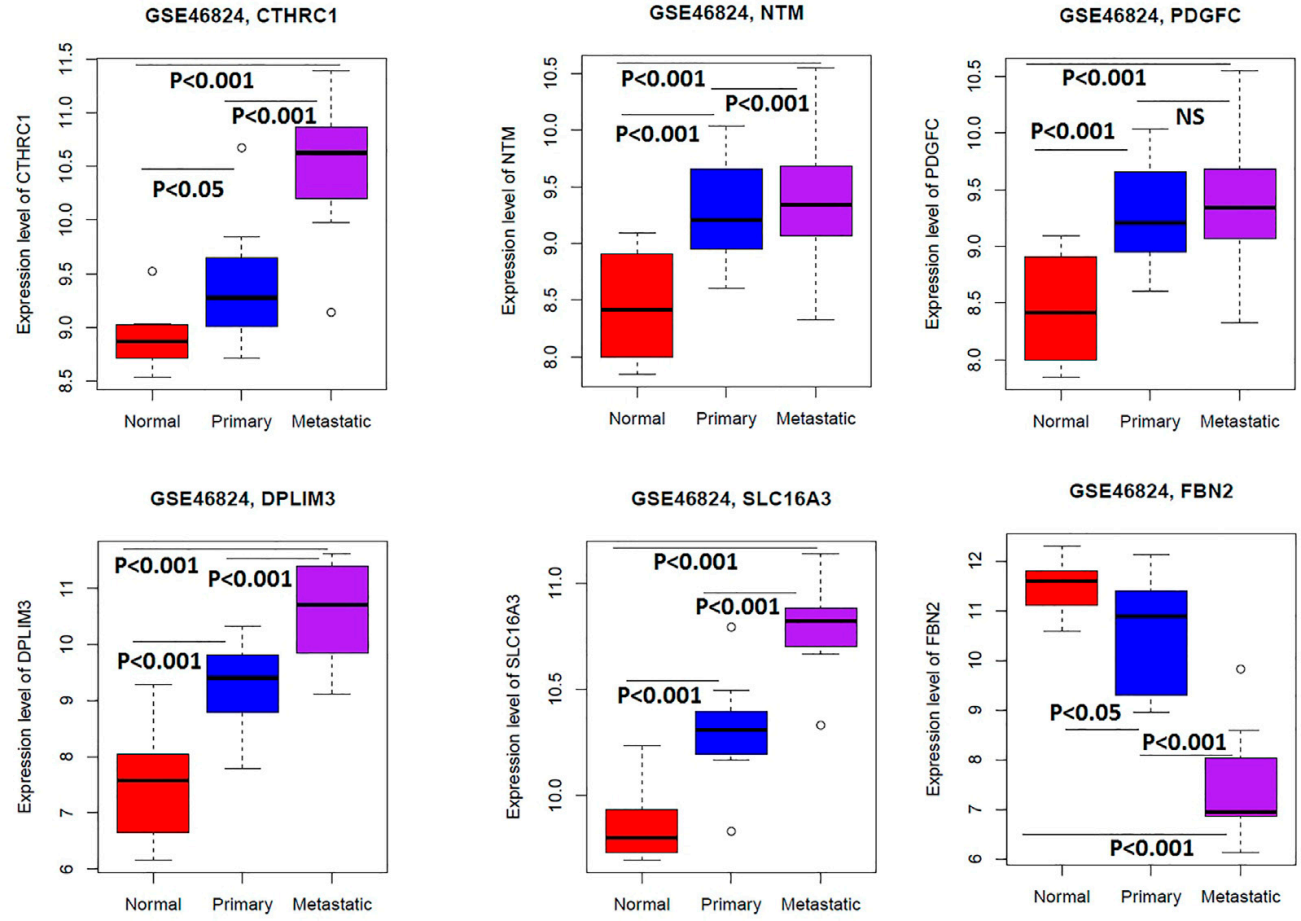
The expression levels of *CTHRC1*, *NTM*, *PDGFC*, *PDLIM3*, *SLC16A3*, and *FBN2* are significantly differentiated among the normal samples, primary tumor, and metastatic tumor of colonic fibroblast (Welch’s t-test, *p* < 0.05). We used the GSE46824 to compare the expression level of genes in the normal samples, primary tumor, and metastatic tumor of colonic fibroblast.

We analysed the expression level differences of five shorter survival-associated genes (*CTHRC1*, *NTM*, *PDGFC*, *PDLIM3*, and *SLC16A3*) and one longer survival-associated gene (*FBN2*) among the normal fibroblast (*n* = 9), primary tumor fibroblast (*n* = 14), and metastatic tumor fibroblast (*n* = 11) samples of GSE46824 [18]. Interestingly, we found that *CTHRC1*, *NTM*, *PDGFC*, *PDLIM3*, and *SLC16A3* are gradually upregulated from normal to a metastatic tumor, indicating the association of these genes in tumor progression (Welch’s t-test, *p* < 0.05) (Figure 7). Besides, the expression of *FBN2* is gradually downregulated from normal to the metastatic tumor, indicating that the downregulation of this gene is associated with tumor progression (Welch’s t-test, *p* < 0.05) (Figure 7). Since the expression levels of *CTHRC1*, *FBN2*, *NTM*, *PDGFC*, *PDLIM3*, and *SLC16A3* are significantly differentiated among the normal samples, primary tumor, and metastatic tumor, we anticipated that these genes are associated with metastasis in colon cancer.

**FIGURE 8:**
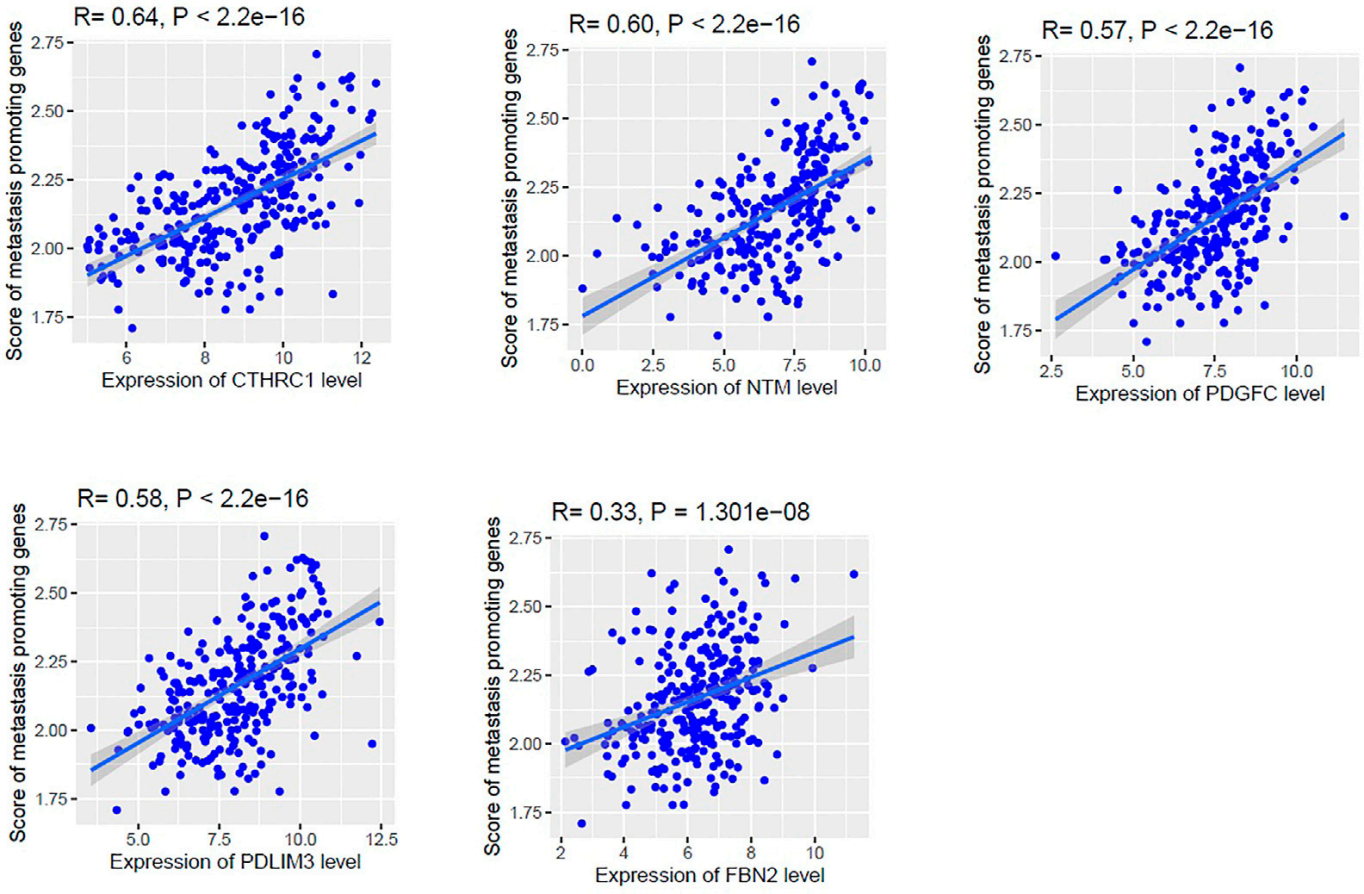
The expression levels of *CTHRC1*, *NTM*, *PDGFC*, *PDLIM3*, and *FBN2* are positively correlated with the ssGSEA scores of metastasis-promoting genes in the TCGA COAD cohort (Spearman’s correlation test, *p* < 0.001).

To prove this hypothesis, we investigated the correlation of these genes with the metastasis-promoting gene set scores. We identified the ssGSEA scores of metastasis-promoting genes and identified the correlation with the expression levels of these six gene signatures (Spearman’s correlation test, *p* < 0.001). We found that the expression levels of *CTHRC1*, *FBN2*, *NTM*, *PDGFC*, and *PDLIM3* are positively correlated with the ssGSEA scores of metastasis-promoting genes in the TCGA COAD cohort (Figure 8).

### Diagnostic Efficacy Evaluation in Fibroblast Dataset and Expression Validation of Key Survival-Associated Genes in Colon Tumor Stroma

**FIGURE 9:**
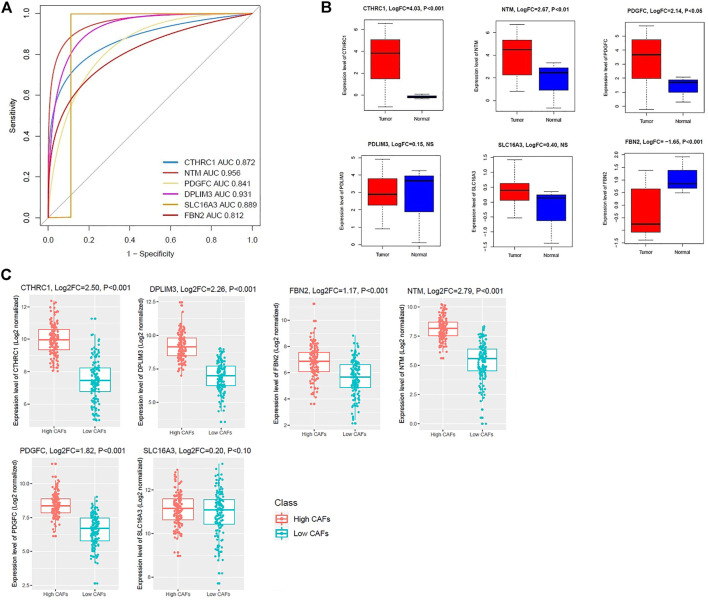
Evaluation of diagnostic efficacy and expression validation of key survival-associated genes. **(A)** The receiver operating characteristic (ROC) curve of survival-associated genes in colonic CAFs and normal fibroblast (GSE46824 dataset). **(B)** Expression validation of shorter survival-associated genes in colon tumor-stromal GSE35602 dataset. NS: Non-significant. **(C)** The shorter survival-associated genes are consistently deregulated in the high-CAFs group versus the low-CAFs group in the TCGA COAD dataset.

We speculate that these six genes (*CTHRC1*, *FBN2*, *NTM*, *PDGFC*, *PDLIM3*, and *SLC16A3*) have diagnostic value in colonic CAFs. We used the GSE46824 dataset to validate our hypothesis, and the results showed that the ROC curve of the expression levels of these six genes (*CTHRC1*, *FBN2*, *NTM*, *PDGFC*, *PDLIM3*, and *SLC16A3*) showed excellent diagnostic value for colonic CAFs and normal colonic fibroblast (Figure 9A). Since CAFs are major regulatory components of tumor stroma [45], we validated the expression levels of survival-associated key genes between colon tumor stroma and normal colon stroma. Interestingly, we found that three upregulated survival-associated genes were also upregulated in colon tumor stroma versus normal colon stroma (*p* < 0.05) (Figure 9B). In addition, downregulated *FBN2* is consistently downregulated in colon tumor stroma (Figure 9B). Moreover, we divided the TCGA COAD samples into the higher-CFAs group versus the lower-CAFs group to identify the expression level of *CTHRC1*, *FBN2*, *NTM*, *PDGFC*, and *PDLIM3*. Interestingly, we revealed that the expression of *CTHRC1*, *FBN2*, *NTM*, *PDGFC*, and *PDLIM3* was highly expressed in the higher-CAFs group (Figure 9C). However, *SLC16A3* is not significantly altered between the higher-CFAs group versus the lower-CAFs group. It indicates that the aberrant expression of CAFs-derived *CTHRC1*, *NTM*, and *PDGFC* is critically involved in the pathogenesis of CRC.

## Discussion

Since CAFs are associated with tumor initiation, invasion, migration, metastasis, and inhibiting immunotherapy response [8–11], identifying key transcriptomes that are correlated with cellular signaling, survival prognosis, immunosuppression, tumor progression, and metastasis should be elucidated. To do this, we identified colonic CAFs-derived transcriptomes which included 72 commonly upregulated (Supplementary Table S2) and 174 commonly downregulated (Supplementary Table S3) DEGs. We used the two datasets, including GSE46824 and GSE70468. The GSE46824 was derived from the patient-derived colon normal fibroblasts and laser micro-dissected CAFs from the surgical colon cancer specimen. On the other hand, the GSE70468 was derived from the primary cultures of patient-derived colon normal fibroblasts (NFs) and cancer-associated fibroblasts (CAFs). We selected the commonly deregulated transcriptomes from both of the datasets (Figures 1C, D), indicating that these transcriptomes are associated with CAFs development in colon cancer tissues, and also involved with the development of CAFs in culture medium. Previous studies showed that CAFs-derived transcriptomes are associated with colon cancer growth, development, and progression [46–50]. For example, the *TNFSF4* gene is associated with epigenetic regulation in colon cancer [49]. Xu et al. reported that *SRGN* can stimulate the metastasis of CRC as a key downstream target of *HIF1-A* [48]. *TGFB2* is frequently mutated in colon cancer and associated with carcinogenesis [50]. The reduced level of *SEPP1* is correlated with M2 macrophage polarization and its downregulation is linked with stemness and proliferation of cells [51]. *AOC3*, another downregulated gene in CAFs, is associated with the enrichment of pathways in colon cancer [52]. Altogether, it indicates that the CAFs derived deregulated transcriptomes are associated with the prognosis of colon cancer.

Then, we investigated the deregulated pathways which are associated with CAFs-derived DEGs. We found that the upregulated DEGs are associated with the enrichment of KEGG pathways that are mainly involved with cancer and cellular signaling and development (Supplementary Table S4). In contrast, CAFs-derived downregulated DEGs are associated with the enrichment of immune regulation and metabolism-associated pathways (Supplementary Table S5). Uddin et al. also found that many of these pathways including pathways in cancer, small cell lung cancer, ECM-receptor interaction, focal adhesion, TGF-beta signaling pathway, and cell adhesion molecules (CAMs) are enriched in colon tumor stroma [53, 54]. It indicates that the CAFs-derived transcriptomes are associated with the stimulation of cancerous and depression of immunological and metabolic pathways in colon cancer.

Since CAFs-derived transcriptomes [4] are crucially related to cancer pathogenesis and cancer therapy [55, 56], we identified key hub genes and survival-associated genes for finding their relationship with survival prognosis, immunosuppression, and metastasis. The PPI-based analysis identified key hub transcriptomes in colonic CAFs. Previous studies consistently found the relevance of these key genes with the pathogenesis of colorectal cancer. For example, the dysregulated FOXM1-PLAUR signaling axis is significantly associated with the progression and metastasis of human colon cancer [57]. In the Rac2^−/−^ mice, a marked reduction in regional colonic lymph node metastasis was found, suggesting a substantial role for Rac2 in controlling spontaneous lymph node metastasis [58]. TGFB2, one of the major members of TGF-β signaling, is critically associated with the progression and susceptibility of colorectal cancer [59]. P Mazzarelli et al. reported that the serum level of *CLU* could represent a diagnostic marker for colon cancer screening [60]. Also, we identified 9 clusters that are associated with the enrichment of KEGG pathways (Table 3). The complement components are associated with the regulation and remodeling of the tumor microenvironment [61]. The chemokines are critically promoting the invasion and metastasis of colorectal cancer [62]. Altogether, PPI-based identification of key hub genes and clusters are involved in the regulation of colon cancer.

The expression level of survival-associated genes is significantly correlated with the shorter survival time of colon cancer patients (Figure 4). The elevated expression of *CTHRC1* correlated with poor prognosis in patients with CRC [63]. It was reported that the overexpression of *SLC16A3* is correlated with prognosis in pancreatic cancer [64]. Collectively, these results indicate that the CAFs-derived genes are associated with the poor prognosis of CRC patients. We selected these survival-associated genes (upregulated *CTHRC1*, *NTM*, *PDGFC*, *PDLIM3*, and *SLC16A3*, and downregulated *FBN2*) for finding the correlations (Spearman’s correlation test, *p* < 0.001) with various immune signatures (Figures 5, 6). Since TAM, macrophages, M2 macrophages, Tregs, T cell exhaustion, and MDSCs are immune suppressive components of TME [45,65,66], the positive correlation of these immune signatures with survival-associated genes indicates the immunosuppressive roles of these genes in the colon TME.

Furthermore, we investigated the expression level of *CTHRC1*, *FBN2*, *NTM*, *PDGFC*, *PDLIM3*, and *SLC16A3* among the normal, primary tumor, and metastatic tumors in a colonic fibroblast dataset and revealed that these genes are gradually deregulated among the three groups, indicating their roles in tumor progression (Welch’s t-test, *p* < 0.05) (Figure 7). Finally, we investigated the correlation of *CTHRC1*, *FBN2*, *NTM*, *PDGFC*, *PDLIM3*, and *SLC16A3* with the metastatic scores in colon cancer data. Interestingly, we found that the expression levels of *CTHRC1*, *FBN2*, *NTM*, *PDGFC*, and *PDLIM3* are positively correlated with the ssGSEA scores of metastatic-promoting genes in colon cancer (Spearman’s correlation test, *p* < 0.001) (Figure 8). Shujuan Ni et al. reported that the elevated level of *CTHRC1* is associated with the progression of CRC [63]. PDGFC is correlated with early diagnosis, cancer grading, and metastatic disease of CRC [67]. In CRC patients with hepatic metastasis, the methylated *FBN2* was detected in the patient’s serum [68]. In summary, these results indicated that the expression of *CTHRC1*, *FBN2*, *NTM*, *PDGFC*, and *PDLIM3* is associated with tumor progression and metastasis of colon cancer cells. Furthermore, we validated the diagnostic efficacy of these key genes in colonic CAFs and found that the *CTHRC1*, *NTM*, *PDGFC*, *PDLIM3*, *SLC16A3*, and *FBN2* genes have diagnostic efficiency in colonic fibroblast (Figure 9A). It indicates that the expression value of these key genes could be used in the diagnostic process. Finally, we validated the expression levels of these key genes in the colon tumor stroma dataset, and we revealed that the *CTHRC1*, *NTM*, *PDGFC*, and *FBN2* genes are consistently altered in colon tumor stroma (Figure 9B). Also, *CTHRC1*, *NTM*, *PDGFC*, and *PDLIM3* are consistently deregulated when compared the high-CAFs group with the low-CAFs group in the TCGA COAD cohort (Figure 9C). It indicates that stromal CAFs-derived transcriptomes may have a crucial contribution to colon cancer. However, the expression levels of *PDLIM3* and *SLC16A3* are not significantly altered in colon tumor stroma (*p* < 0.05) and the expression level of *FBN2* showed the opposite trend in the high-CAFs group (Figure 9C). As far as we know, *FBN2* is a protein-coding gene, which is associated with tissue microfibrils and may be involved in elastic fiber assembly [69]. It has been reported that FBN2 could have excellent diagnostic value for smooth muscle sarcoma and rhabdomyosarcoma [70, 71]. Besides, it also has been proved that *FBN2* might both have tumor-suppressive effects and is a typical basement membrane marker in several types of cancers [72]. Previous studies have found that FBN2 harbour cancer-specific promoter methylation in human colorectal cancer [73]. Furthermore, clinical research has found that FBN2 gene methylation exhibited in 63% of tumor samples of patients with primary colorectal carcinoma, and FBN2 might be an early and frequent event in precancerous and cancerous lesions of the colon and rectum [68]. Although little is known about FBN2 biological function regarding epigenetic changes in human cancers, the methylation of these genes has great potential to detect early-stage colon cancer [74]. FBN2 annotation research showed that high expression of FBN2 was mainly enriched in extracellular matrix (ECM) receptor interaction and epithelial-mesenchymal transition (EMT) pathway [75]. In contrast, high expression of FBN2 was found as a risk factor in lung and gastric cancer [76, 77]. In our research, we found that FBN2 is highly expressed in normal fibroblasts, and downregulated FBN2 is consistently correlated with the shorter survival of colon cancer patients, this result gives rise to the idea to study the role of FBN2 in a deeper way. Since we divided COAD samples into the high-CAFs group versus the low-CAFs group and found a higher level of *FBN2* in the high-CAFs group, suggesting that the tumor cell contributed to the higher expression of *FBN2* but stromal CAFs contributed to the lower expression level of *FBN2*. Furthermore, in the TCGA COAD cohort, we found that the expression level of *FBN2* is associated with the infiltrations of immune cells (Figure 6), indicating that tumor cells contributed to the higher expression level of *FBN2* and stromal CAFs contributed to the lower level of *FBN*2, which ultimately associated with lowering survival time and increasing the immune infiltrations in colon cancer patients.

Altogether, these results indicate the expression of CAFs-derived key transcriptomes (*CTHRC1*, *NTM*, and *PDGFC*) is associated with poor survival prognosis, immunosuppression, tumor progression, and metastasis in human colon cancer. Numerous evidence indicated that immunohistochemistry is a highly effective substantial tool to predict survival prognosis in patients with various cancer types [78]. Since laser-capture micro-dissected stromal FFPE tissue can be utilized to identify molecular proteomic and transcriptomic profiling [79], measuring the key genes with immunohistochemistry in the laser micro-dissected CAFs may lead the clinicians to taking decisions in diagnosis and specific gene-oriented targeted therapy of colon cancer patients. However, for using these findings in clinical and therapeutic applications, further experimental and clinical verification would be necessary.

## Conclusion

The identification of colonic CAFs-derived key transcriptomes may provide insight into the association of these key genes with survival prognosis, immunosuppression, tumor progression, and metastasis.

## Data Availability

The GEO datasets GSE46824, GSE70468, GSE17536, and GSE35602 were used in this study and are available in The National Biotechnology Information Centre Gene (NCBI-Gene) database (https://www.ncbi.nlm.nih.gov/gene). The TCGA-COAD cohort was downloaded from The Cancer Genome Atlas (TCGA) database (https://portal.gdc.cancer.gov/).
