# Supplementary material for: Cancer-Associated Stromal Fibroblast-Derived Transcriptomes Predict Poor Clinical Outcomes and Immunosuppression in Colon Cancer
Source: Pathol Oncol Res. 2022 Aug 4;28:1610350. doi: 10.3389/pore.2022.1610350 (PMC9385976; doi:10.3389/pore.2022.1610350)
Supplement: Supplementary file 1 [file Table5.pdf]

Supplementary Table S5. The list of KEGG pathways associated with downregulated DEGs in colonic CAFs

**KEGG pathways**	#Genes in Overlap (k)	k/K	P-value
Complement and coagulation cascades	7	0.1014	2.13E-08
Glycolysis / Gluconeogenesis	6	0.0968	2.98E-07
Vascular smooth muscle contraction	7	0.0609	7.32E-07
Fatty acid metabolism	5	0.119	1.06E-06
Chemokine signaling pathway	8	0.0423	1.83E-06
GnRH signaling pathway	6	0.0594	5.35E-06
Pathways in cancer	9	0.0277	1.31E-05
Tyrosine metabolism	4	0.0952	3.30E-05
Progesterone-mediated oocyte maturation	5	0.0588	3.51E-05
Dilated cardiomyopathy	5	0.0556	4.62E-05
Gap junction	5	0.0556	4.62E-05
Purine metabolism	6	0.0377	7.01E-05
beta-Alanine metabolism	3	0.1364	1.15E-04
Calcium signaling pathway	6	0.0337	1.30E-04
Focal adhesion	6	0.0302	2.38E-04
Metabolism of xenobiotics by cytochrome P450	4	0.0571	2.46E-04
Histidine metabolism	3	0.1034	2.66E-04
Insulin signaling pathway	5	0.0365	3.34E-04
Propanoate metabolism	3	0.0909	3.92E-04
Pyruvate metabolism	3	0.075	6.94E-04
Limonene and pinene degradation	2	0.2	8.15E-04
Melanogenesis	4	0.0396	9.88E-04
MAPK signaling pathway	6	0.0225	1.12E-03
NOD-like receptor signaling pathway	3	0.0484	2.48E-03
Retinol metabolism	3	0.0469	2.71E-03
Adipocytokine signaling pathway	3	0.0448	3.09E-03
Long-term depression	3	0.0429	3.50E-03
Drug metabolism - cytochrome P450	3	0.0417	3.79E-03
Ascorbate and aldarate metabolism	2	0.08	5.21E-03
ECM-receptor interaction	3	0.0357	5.83E-03
Small cell lung cancer	3	0.0357	5.83E-03
Cytokine-cytokine receptor interaction	5	0.0189	6.03E-03
Butanoate metabolism	2	0.0588	9.49E-03
Tryptophan metabolism	2	0.05	1.30E-02
Oocyte meiosis	3	0.0265	1.31E-02
ABC transporters	2	0.0455	1.56E-02
Lysine degradation	2	0.0455	1.56E-02
Valine, leucine and isoleucine degradation	2	0.0455	1.56E-02
Vasopressin-regulated water reabsorption	2	0.0455	1.56E-02
Neurotrophin signaling pathway	3	0.0238	1.75E-02
Type II diabetes mellitus	2	0.0426	1.76E-02
Axon guidance	3	0.0233	1.86E-02

Glycerolipid metabolism	2	0.0408	1.91E-02
Amyotrophic lateral sclerosis (ALS)	2	0.0377	2.21E-02
Systemic lupus erythematosus	3	0.0216	2.26E-02
Arginine and proline metabolism	2	0.037	2.29E-02
Vibrio cholerae infection	2	0.037	2.29E-02
Basal cell carcinoma	2	0.0364	2.37E-02
Steroid hormone biosynthesis	2	0.0364	2.37E-02
Arachidonic acid metabolism	2	0.0345	2.61E-02
Colorectal cancer	2	0.0323	2.96E-02
Melanoma	2	0.0282	3.79E-02
Arrhythmogenic right ventricular cardiomyopathy (ARVC	2	0.027	4.09E-02
Fc epsilon RI signaling pathway	2	0.0253	4.60E-02
