# Supplementary material for: Cancer-Associated Stromal Fibroblast-Derived Transcriptomes Predict Poor Clinical Outcomes and Immunosuppression in Colon Cancer
Source: Pathol Oncol Res. 2022 Aug 4;28:1610350. doi: 10.3389/pore.2022.1610350 (PMC9385976; doi:10.3389/pore.2022.1610350)
Supplement: Supplementary file 2 [file Table4.pdf]

Supplementary Table S4. The list of KEGG pathways associated with upregulated DEGs in colonic CAFs

KEGG pathways	Genes in Overlap (k)	k/K	P-value
Arrhythmogenic right ventricular cardiomyopathy (ARVC)	5	0.0676	2.15E-07
Regulation of actin cytoskeleton	6	0.0282	2.18E-06
Colorectal cancer	3	0.0484	1.84E-04
Pancreatic cancer	3	0.0429	2.63E-04
Pathways in cancer	5	0.0154	2.78E-04
TGF-beta signaling pathway	3	0.0349	4.82E-04
Cytokine-cytokine receptor interaction	4	0.0151	1.26E-03
Pathogenic Escherichia coli infection	2	0.0357	4.43E-03
Focal adhesion	3	0.0151	5.29E-03
p53 signaling pathway	2	0.0294	6.46E-03
Complement and coagulation cascades	2	0.029	6.65E-03
Melanoma	2	0.0282	7.03E-03
Adherens junction	2	0.0274	7.41E-03
Chronic myeloid leukemia	2	0.0274	7.41E-03
Hypertrophic cardiomyopathy (HCM)	2	0.0241	9.49E-03
Small cell lung cancer	2	0.0238	9.71E-03
Hematopoietic cell lineage	2	0.023	1.04E-02
Prostate cancer	2	0.0225	1.09E-02
Dilated cardiomyopathy	2	0.0222	1.11E-02
Gap junction	2	0.0222	1.11E-02
MAPK signaling pathway	3	0.0112	1.18E-02
Leukocyte transendothelial migration	2	0.0172	1.79E-02
Cell cycle	2	0.016	2.06E-02
Axon guidance	2	0.0155	2.19E-02
Cell adhesion molecules (CAMs)	2	0.015	2.31E-02
Wnt signaling pathway	2	0.0132	2.93E-02
Endocytosis	2	0.011	4.07E-02
Chemokine signaling pathway	2	0.0106	4.40E-02
