# Supplementary material for: Cancer-Associated Stromal Fibroblast-Derived Transcriptomes Predict Poor Clinical Outcomes and Immunosuppression in Colon Cancer
Source: Pathol Oncol Res. 2022 Aug 4;28:1610350. doi: 10.3389/pore.2022.1610350 (PMC9385976; doi:10.3389/pore.2022.1610350)
Supplement: Supplementary file 3 [file Table1.pdf]

Supplementary Table S1. The list of immune markers for identifying ssGSEA scores

CD4+ Regulatory T cells	CD8+ T cells	Macrophages	NK cells	Treg genes	Metastasis-promoting genes	T cell exhaustion	TAM	M2 Macrophage	MDSCs	CAFs
C15orf53	CD8A	C11orf45	KLRC1	IL12RB2	CYBC1	CXCL13	IL10	VSIG4	PARVG	POSTN
CTLA4		CD68	KLRF1	TMPRSS6	BACH2	LAYN	CCL2	MS4A4A	ITGAL	COL1A1
FOXP3		CLEC5A		CTSC	SPNS2	CTLA4	CD68	CD163	PTGER2	COL1A2
GPR15		CYBB		LAPTM4B	FBXO7	TIGIT			PTGES2	COL6A1
IL32		FUCA1		TFRC	GRSF1	LAG3			S100A8	COL6A2
IL4		GPNMB		RNF145	LRIG1	PDCD1			PSAP	COL6A3
IL5		HS3ST2		NETO2	ARHGEF1	HAVCR2			FERMT3	CSPG4
		LGMN		ADAT2	NBEAL2				GPSM3	DCN
		MMP9		CHST2	CYBA				S100A9	DES
		TM4SF19		CTLA4	HSP90AA1				ITGAM	FAP
				NFE2L3	TBC1D22A				CD86	TNC
				LIMA1	CYBB				IL18BP	PDGFRA
				IL1R2	FAM175B				FCGR2B	PDGFRB
				ICOS	NCF2				CXCR4	ACTA2
				HSDL2	ENTPD1				IL4R	S100A4
				HTATIP2					CCR2	THY1
				FKBP1A					CD2	VIM
				TIGIT					FCGR2A	
				CCR8					FCGR3A	
				LTA					CD14	
				SLC35F2						
				IL21R						
				AHCYL1						
				SOCS2						
				ETV7						
				BCL2L1						
				RRAGB						
				ACSL4						
				CHRNA6						
				BATF						
				LAX1						
				ADPRH						
				TNFRSF4						
				ANKRD10						
				CD274						
				CASP1						
				LY75						
				NPTN						
				SSTR3						
				GRSF1						
				CSF2RB						

TMEM184C
NDFIP2
ZBTB38
ERI1
TRAF3
NAB1
HS3ST3B1
LAYN
JAK1
VDR
LEPROT
GCNT1
PTPRJ
IKZF2
CSF1
ENTPD1
TNFRSF18
METTL7A
KSR1
SSH1
CADM1
IL1R1
ACP5
CHST7
THADA
CD177
NFAT5
ZNF282
MAGEH1
