# Supplementary material for: Cancer-Associated Stromal Fibroblast-Derived Transcriptomes Predict Poor Clinical Outcomes and Immunosuppression in Colon Cancer
Source: Pathol Oncol Res. 2022 Aug 4;28:1610350. doi: 10.3389/pore.2022.1610350 (PMC9385976; doi:10.3389/pore.2022.1610350)
Supplement: Supplementary file 4 [file Table2.pdf]

Supplementary Table S2. The list of common 72 upregulated DEGs in colonic CAFs						
**Entrez Gene ID**	**Gene symbol**	**Gene name**	**LogFC (GSE46824)**	**P value (GSE46824)**	**LogFC (GSE70468)**	**P value (GSE70468)**
3675	*ITGA3*	integrin subunit alpha 3	1.030	2.17E-04	1.088	2.43E-02
51176	*LEF1*	lymphoid enhancer binding factor 1	1.048	3.38E-02	2.486	4.04E-03
1829	*DSG2*	desmoglein 2	1.113	3.43E-02	0.709	9.59E-04
1832	*DSP*	desmoplakin	1.960	5.31E-03	1.388	4.94E-02
5318	*PKP2*	plakophilin 2	1.461	1.02E-04	0.918	1.84E-02
5880	*RAC2*	Rac family small GTPase 2	1.022	8.65E-05	0.809	4.43E-02
56034	*PDGFC*	platelet derived growth factor C	0.783	1.94E-04	1.040	6.48E-04
2149	*F2R*	coagulation factor II thrombin receptor	1.187	4.02E-03	1.478	5.37E-03
7074	*TIAM1*	TIAM Rac1 associated GEF 1	0.940	9.72E-04	0.681	2.15E-02
81624	*DIAPH3*	diaphanous related formin 3	1.174	3.67E-04	0.752	2.17E-03
7042	*TGFB2*	transforming growth factor beta 2	2.305	3.57E-09	0.594	1.35E-02
1021	*CDK6*	cyclin dependent kinase 6	0.790	4.21E-05	0.830	2.58E-03
10468	*FST*	follistatin	1.299	2.89E-03	0.873	2.38E-02
4052	*LTBP1*	latent transforming growth factor beta binding protein 1	0.631	3.93E-02	0.619	3.89E-02
3575	*IL7R*	interleukin 7 receptor	1.414	7.48E-04	1.879	1.85E-03
7292	*TNFSF4*	TNF superfamily member 4	3.485	1.14E-08	1.778	4.61E-02
10376	*TUBA1B*	tubulin alpha 1b	0.783	3.13E-04	0.781	4.30E-02
9076	*CLDN1*	claudin 1	1.259	5.24E-03	0.760	3.40E-02
5366	*PMAIP1*	phorbol-12-myristate-13-acetate-induced protein 1	0.878	3.00E-03	1.549	6.84E-04
5329	*PLAUR*	plasminogen activator, urokinase receptor	0.680	3.10E-03	0.979	1.28E-02
4908	*NTF3*	neurotrophin 3	1.654	2.75E-11	2.003	1.90E-03
1948	*EFNB2*	ephrin B2	1.799	1.52E-05	1.359	1.11E-02
23705	*CADM1*	cell adhesion molecule 1	0.699	8.96E-03	2.275	2.03E-03
30845	*EHD3*	EH domain containing 3	0.633	9.29E-03	0.704	1.21E-03
2151	*F2RL2*	coagulation factor II thrombin receptor like 2	1.049	1.29E-02	2.212	2.87E-03

27338	*UBE2S*	ubiquitin conjugating enzyme E2 S	0.612	3.06E-04	0.877	2.67E-03
94274	*PPP1R14A*	protein phosphatase 1 regulatory inhibitor subunit 14A	1.011	7.42E-05	1.368	2.60E-02
1839	*HBEGF*	heparin binding EGF like growth factor	0.910	1.32E-02	0.664	3.52E-02
55790	*CSGALNACT 1*	chondroitin sulfate N-acetylgalactosaminyltra nsferase 1	1.324	9.63E-04	1.011	3.45E-03
10085	*EDIL3*	EGF like repeats and discoidin domains 3	1.269	4.14E-04	1.771	1.55E-03
1012	*CDH13*	cadherin 13	1.791	1.32E-05	1.039	1.37E-02
10611	*PDLIM5*	PDZ and LIM domain 5	0.826	2.53E-04	0.613	1.86E-02
115908	*CTHRC1*	collagen triple helix repeat containing 1	0.937	7.85E-04	1.054	4.63E-03
116372	*LYPD1*	LY6/PLAUR domain containing 1	0.630	4.18E-02	1.005	4.48E-02
133418	*EMB*	embigin	1.980	8.39E-06	0.631	3.57E-02
144406	*WDR66*	WD repeat domain 66	0.884	8.18E-04	0.621	1.98E-03
23022	*PALLD*	palladin, cytoskeletal associated protein	0.946	1.20E-08	0.713	3.37E-03
2307	*FOXS1*	forkhead box S1	0.595	4.14E-03	1.314	4.39E-02
2313	*FLI1*	Fli-1 proto-oncogene, ETS transcription factor	1.362	1.73E-07	0.915	5.12E-05
25907	*TMEM158*	transmembrane protein 158	0.608	1.37E-03	1.222	3.80E-03
26037	*SIPA1L1*	signal induced proliferation associated 1 like 1	0.786	1.73E-04	0.995	1.14E-03
27132	*CPNE7*	copine 7	0.769	6.81E-05	1.672	2.41E-03
27295	*PDLIM3*	PDZ and LIM domain 3	2.278	1.13E-06	1.710	4.51E-02
353322	*ANKRD37*	ankyrin repeat domain 37	1.061	1.05E-03	1.618	1.92E-02
3835	*KIF22*	kinesin family member 22	0.832	3.30E-03	0.690	4.55E-02
3856	*KRT8*	keratin 8	1.343	6.56E-04	0.640	1.59E-02
3880	*KRT19*	keratin 19	1.339	5.20E-04	1.947	4.44E-02
4493	*MT1E*	metallothionein 1E	1.584	4.91E-03	1.058	3.29E-02
4499	*MT1M*	metallothionein 1M	1.633	6.94E-03	0.764	3.62E-02
4603	*MYBL1*	MYB proto-oncogene like 1	1.094	1.15E-02	1.209	2.48E-02
50863	*NTM*	neurotrimin	1.899	1.13E-06	1.079	1.02E-02

5157	*PDGFRL*	platelet derived growth factor receptor like	1.014	8.45E-06	0.814	4.14E-02
5552	*SRGN*	serglycin	2.531	1.40E-03	1.359	2.29E-02
56243	*KIAA1217*	KIAA1217	1.177	1.10E-05	1.195	3.92E-03
56256	*SERTAD4*	SERTA domain containing 4	1.381	1.45E-04	1.016	4.91E-03
56662	*VTRNA1-3*	vault RNA 1-3	1.122	4.71E-03	0.730	5.84E-03
57528	*KCTD16*	potassium channel tetramerization domain containing 16	1.445	6.22E-07	0.799	1.19E-03
6461	*SHB*	SH2 domain containing adaptor protein B	0.598	3.30E-07	0.829	2.75E-04
64943	*NT5DC2*	5'-nucleotidase domain containing 2	0.625	5.85E-04	0.649	1.23E-02
6840	*SVIL*	supervillin	0.810	2.28E-02	0.997	1.96E-02
7262	*PHLDA2*	pleckstrin homology like domain family A member 2	0.936	1.48E-06	0.934	4.13E-02
79933	*SYNPO2L*	synaptopodin 2 like	1.272	3.67E-05	1.099	3.30E-02
84419	*C15orf48*	chromosome 15 open reading frame 48	2.259	2.18E-03	1.483	5.30E-03
84561	*SLC12A8*	solute carrier family 12 member 8	0.647	6.99E-03	1.296	2.45E-02
84679	*SLC9A7*	solute carrier family 9 member A7	1.068	6.41E-05	1.022	2.72E-02
864	*RUNX3*	RUNX family transcription factor 3	0.736	1.55E-07	0.604	4.95E-04
8870	*IER3*	immediate early response 3	0.723	3.36E-03	0.887	1.18E-02
9123	*SLC16A3*	solute carrier family 16 member 3	0.681	1.71E-07	0.961	8.14E-04
9262	*STK17B*	serine/threonine kinase 17b	1.092	1.89E-04	0.754	1.62E-03
9603	*NFE2L3*	nuclear factor, erythroid 2 like 3	0.729	3.80E-07	1.555	1.60E-05
9891	*NUAK1*	NUAK family kinase 1	0.751	2.16E-03	1.148	4.41E-02
157285	*SGK223*	homolog of rat pragma of Rnd2	0.756	1.73E-04	1.789	1.81E-02
