# Supplementary material for: Cancer-Associated Stromal Fibroblast-Derived Transcriptomes Predict Poor Clinical Outcomes and Immunosuppression in Colon Cancer
Source: Pathol Oncol Res. 2022 Aug 4;28:1610350. doi: 10.3389/pore.2022.1610350 (PMC9385976; doi:10.3389/pore.2022.1610350)
Supplement: Supplementary file 5 [file Table6.pdf]

Supplementary Table S6. List of hub genes in the PPI network of DEGs ranked by degree method

Rank	Name of genes	Degree of intereaction
1	CLU	18
1	CXCL12	18
3	VTN	15
4	ADCY3	13
5	A2M	12
5	LDLR	12
5	TGFB2	12
5	PPARG	12
5	ADCY9	12
5	PLAUR	12
11	ADCY4	11
11	PTK2B	11
13	RAC2	10
13	CFD	10
13	BIN1	10
16	SLC2A4	9
16	LEF1	9
18	SRGN	8
18	VLDLR	8
18	PROS1	8
18	DSP	8
18	GUCY1B3	8
18	IL7R	8
18	DSG2	8
25	ISLR	7
25	FGF13	7
25	ABCA1	7
25	PDE3B	7
25	CCL13	7
25	GUCY1A3	7
25	PTGER3	7
25	ADORA1	7
25	HMOX1	7
25	HBEGF	7
35	PKP2	6
35	EDIL3	6
35	EFNB2	6
35	C1R	6
35	C1S	6
35	ALDH2	6
35	ALDH3A2	6
35	GCH1	6
35	TNFRSF1B	6
35	BCL6	6

35	ITGA3	6
46	RBP4	5
46	NTF3	5
46	IL33	5
46	KRT19	5
46	KRT8	5
46	STOX2	5
46	F2R	5
46	FBLN1	5
46	FST	5
46	LTBP1	5
46	LYPD1	5
46	FZD1	5
46	MAPK10	5
46	RUNX3	5
60	RCAN2	4
60	KCNE3	4
60	CADM1	4
60	TIAM1	4
60	FGF7	4
60	DPP4	4
60	IL1R1	4
60	ACSS1	4
60	PPL	4
60	ADM	4
60	F2RL2	4
60	GNA14	4
60	ADH1A	4
60	ADH1C	4
60	ADH1B	4
60	BCL2	4
60	CTSO	4
60	TLE1	4
60	TNFSF4	4
79	CACNB2	3
79	CLDN1	3
79	SMOC2	3
79	SLIT3	3
79	CYP39A1	3
79	PDLIM3	3
79	LPIN1	3
79	ZEB2	3
79	TMTC2	3
79	SEMA3B	3
79	EML1	3
79	ATP8B4	3
79	PGRMC1	3

79	MT1E	3
79	EPB41L3	3
79	C2	3
95	MASP1	2
95	KIAA1217	2
95	PBX1	2
95	CDH13	2
95	TLE2	2
95	NFASC	2
95	CCL8	2
95	LYNX1	2
95	CHRDL1	2
95	GREM2	2
95	CLIC2	2
95	GABRE	2
95	PDLIM5	2
95	TIMP4	2
95	PRKCH	2
95	PLA2G2A	2
95	RASSF2	2
95	STK17B	2
95	LSS	2
95	PTPRD	2
95	MOXD1	2
95	OSR2	2
95	SLC16A3	2
95	TUBA1B	2
95	SIPA1L1	2
95	PPP1R3C	2
95	FBN2	2
95	AOC3	2
95	ANKRD37	2
95	TGFBR3	2
95	OLFML3	2
95	APBB1IP	2
95	RSPO2	2
95	RSPO3	2
95	GALNT12	2
95	GLI3	2
95	SHB	2
95	EHD3	2
95	LAMA2	2
95	KIAA0513	2
95	LAMA3	2
95	KCND2	2
95	KCNK1	2
138	KCTD16	1

138	SMPDL3A	1
138	CDK6	1
138	NTM	1
138	WEE1	1
138	NOVA1	1
138	MFSD6	1
138	MT1M	1
138	PCSK6	1
138	PPP4R4	1
138	PALLD	1
138	SVIL	1
138	PAMR1	1
138	SRPX	1
138	PREB	1
138	EMILIN2	1
138	CTHRC1	1
138	SYNPO2L	1
138	PXK	1
138	SLC25A27	1
138	SLC2A12	1
138	SLC43A2	1
138	TDRD6	1
138	TNFAIP8	1
138	DIAPH3	1
138	DOK6	1
138	UBE2S	1
138	EYA4	1
138	ATF5	1
138	EBF3	1
138	PDZRN3	1
138	EMB	1
138	ENPP5	1
138	FAM149A	1
138	AKR1C1	1
138	FLI1	1
138	FOXS1	1
138	ANKRD29	1
138	IL17RD	1
138	ST6GALNAC3	1
138	PMAIP1	1
138	LIN7A	1
138	HSD11B1	1
138	TBC1D2B	1
138	C15orf48	1
138	C15orf59	1
