# Supplementary material for: Cancer-Associated Stromal Fibroblast-Derived Transcriptomes Predict Poor Clinical Outcomes and Immunosuppression in Colon Cancer
Source: Pathol Oncol Res. 2022 Aug 4;28:1610350. doi: 10.3389/pore.2022.1610350 (PMC9385976; doi:10.3389/pore.2022.1610350)
Supplement: Supplementary file 6 [file Table3.pdf]

Supplementary Table S3. The list of common 174 downregulated DEGs in colonic CAFs						
**Entrez Gene ID**	**Gene symbol**	**Gene name**	**LogFC (GSE46824)**	**P Value (GSE46824)**	**LogFC (GSE70468)**	**P Value (GSE70468)**
715	*C1R*	complement C1r	-0.873	5.49E-04	-0.915	3.98E-02
716	*C1S*	complement C1s	-0.873	1.84E-03	-0.824	9.64E-03
717	*C2*	complement C2	-0.920	3.71E-02	-1.609	9.12E-03
1675	*CFD*	complement factor D	-2.402	6.34E-08	-3.601	1.20E-03
2	*A2M*	alpha-2-macroglobulin	-1.939	2.15E-03	-2.128	8.36E-03
5627	*PROS1*	protein S	-0.897	1.89E-04	-0.854	5.02E-03
5648	*MASP1*	mannan binding lectin serine peptidase 1	-1.437	5.79E-06	-1.253	5.32E-03
124	*ADH1A*	alcohol dehydrogenase 1A (class I), alpha polypeptide	-1.816	4.90E-11	-3.536	1.93E-03
125	*ADH1B*	alcohol dehydrogenase 1B (class I), beta polypeptide	-4.255	2.84E-10	-2.711	8.93E-04
126	*ADH1C*	alcohol dehydrogenase 1C (class I), gamma polypeptide	-1.617	1.84E-05	-3.895	3.00E-03
217	*ALDH2*	aldehyde dehydrogenase 2 family member	-1.239	5.41E-05	-0.709	4.58E-02
224	*ALDH3A2*	aldehyde dehydrogenase 3 family member A2	-0.596	1.15E-04	-0.824	5.78E-03
84532	*ACSS1*	acyl-CoA synthetase short chain family member 1	-0.894	4.20E-05	-0.909	1.43E-02
109	*ADCY3*	adenylate cyclase 3	-0.784	3.35E-05	-0.764	3.98E-03
115	*ADCY9*	adenylate cyclase 9	-0.729	2.83E-03	-0.976	4.91E-04
196883	*ADCY4*	adenylate cyclase 4	-0.665	7.70E-04	-1.206	2.99E-03
5320	*PLA2G2A*	phospholipase A2 group IIA	-1.937	3.74E-06	-0.753	1.49E-02
2982	*GUCY1A3*	guanylate cyclase 1 soluble subunit alpha 3	-2.194	1.71E-04	-1.974	3.06E-02
2983	*GUCY1B1*	guanylate cyclase 1 soluble subunit beta 1	-1.432	1.47E-03	-1.057	2.61E-02
5583	*PRKCH*	protein kinase C eta	-1.976	1.36E-08	-1.080	1.07E-03
2185	*PTK2B*	protein tyrosine kinase 2 beta	-0.745	1.73E-04	-0.656	1.20E-02
53358	*SHC3*	SHC adaptor protein 3	-1.121	5.38E-05	-1.029	1.48E-02
6355	*CCL8*	C-C motif chemokine ligand 8	-1.033	2.14E-02	-1.680	9.56E-04
6357	*CCL13*	C-C motif chemokine ligand 13	-1.189	8.55E-03	-1.887	1.83E-02
6387	*CXCL12*	C-X-C motif chemokine ligand 12	-1.425	4.25E-03	-3.208	1.09E-03

5602	*MAPK10*	mitogen-activated protein kinase 10	-1.039	1.01E-04	-0.828	6.68E-03
3908	*LAMA2*	laminin subunit alpha 2	-0.712	9.10E-03	-1.108	1.72E-02
3909	*LAMA3*	laminin subunit alpha 3	-1.658	6.59E-04	-1.238	2.34E-03
596	*BCL2*	BCL2 apoptosis regulator	-1.658	6.59E-04	-1.238	2.34E-03
8321	*FZD1*	frizzled class receptor 1	-0.960	1.70E-07	-0.597	2.41E-02
2252	*FGF7*	fibroblast growth factor 7	-1.388	7.81E-03	-0.809	3.12E-02
2258	*FGF13*	fibroblast growth factor 13	-1.023	1.89E-08	-1.361	7.20E-03
2737	*GLI3*	GLI family zinc finger 3	-1.063	1.63E-04	-0.748	7.43E-03
5468	*PPARG*	peroxisome proliferator activated receptor gamma	-1.600	1.18E-05	-1.465	1.16E-02
8639	*AOC3*	amine oxidase copper containing 3	-3.151	4.11E-08	-3.748	7.01E-04
5140	*PDE3B*	phosphodiesterase 3B	-1.047	1.83E-04	-0.774	7.92E-03
783	*CACNB2*	calcium voltage-gated channel auxiliary subunit beta 2	-1.707	4.21E-05	-0.713	4.96E-04
5733	*PTGER3*	prostaglandin E receptor 3	-1.144	3.02E-02	-1.175	4.37E-02
9630	*GNA14*	G protein subunit alpha 14	-2.187	4.46E-08	-0.637	1.51E-02
7448	*VTN*	vitronectin	-1.446	1.78E-04	-1.091	3.18E-03
1645	*AKR1C1*	aldo-keto reductase family 1 member C1	-2.269	1.03E-05	-0.812	2.24E-02
443	*ASPA*	aspartoacylase	-2.055	4.36E-06	-2.050	6.23E-04
6517	*SLC2A4*	solute carrier family 2 member 4	-0.601	1.39E-06	-0.620	1.12E-02
5507	*PPP1R3C*	protein phosphatase 1 regulatory subunit 3C	-0.604	1.65E-03	-0.851	2.88E-02
3554	*IL1R1*	interleukin 1 receptor type 1	-0.833	8.73E-04	-0.933	6.78E-03
7133	*TNFRSF1B*	TNF receptor superfamily member 1B	-0.620	1.18E-02	-0.652	6.81E-03
19	*ABCA1*	ATP binding cassette subfamily A member 1	-0.778	6.00E-04	-0.701	9.45E-03
23461	*ABCA5*	ATP binding cassette subfamily A member 5	-1.220	5.69E-07	-0.671	7.35E-03
6586	*SLIT3*	slit guidance ligand 3	-1.381	3.19E-04	-1.562	2.20E-02
7869	*SEMA3B*	semaphorin 3B	-0.726	9.52E-03	-0.956	4.67E-02
3290	*HSD11B1*	hydroxysteroid 11-beta dehydrogenase 1	-0.791	2.14E-02	-0.927	3.34E-02
2053	*EPHX2*	epoxide hydrolase 2	-1.380	6.85E-07	-0.888	1.67E-03
23136	*EPB41L3*	erythrocyte membrane protein band 4.1 like 3	-1.118	2.09E-04	-1.038	1.02E-02
134	*ADORA1*	adenosine A1 receptor	-1.436	8.71E-08	-1.109	4.99E-03

2564	*GABRE*	gamma-aminobutyric acid type A receptor subunit epsilon	-1.083	4.76E-04	-1.129	1.70E-04
3949	*LDLR*	low density lipoprotein receptor	-0.675	9.72E-03	-0.648	3.43E-02
23114	*NFASC*	neurofascin	-1.070	1.91E-03	-1.643	1.52E-04
7465	*WEE1*	WEE1 G2 checkpoint kinase	-0.624	4.30E-02	-0.700	1.09E-03
1519	*CTSO*	cathepsin O	-0.609	7.98E-05	-0.797	4.17E-03
3613	*IMPA2*	inositol monophosphatase 2	-1.010	2.35E-03	-0.641	6.71E-03
117178	*SSX2IP*	SSX family member 2 interacting protein	-1.350	3.53E-03	-0.716	2.97E-02
90865	*IL33*	interleukin 33	-1.952	4.80E-04	-2.756	3.41E-04
3162	*HMOX1*	heme oxygenase 1	-0.830	1.91E-05	-1.053	4.34E-02
79695	*GALNT12*	polypeptide N-acetylgalactosaminyltransferase 12	-0.730	1.05E-02	-0.886	2.62E-02
4047	*LSS*	lanosterol synthase	-0.660	2.26E-03	-0.682	1.55E-02
51302	*CYP39A1*	cytochrome P450 family 39 subfamily A member 1	-2.782	2.43E-04	-0.878	9.04E-03
256435	*ST6GALNAC3*	ST6 N-acetylgalactosaminide alpha-2,6-sialyltransferase 3	-1.132	3.86E-07	-0.993	6.12E-03
2643	*GCH1*	GTP cyclohydrolase 1	-0.731	1.79E-03	-0.941	5.67E-05
10008	*KCNE3*	potassium voltage-gated channel subfamily E regulatory subunit 3	-1.195	3.00E-07	-1.347	8.72E-03
10113	*PREB*	prolactin regulatory element binding	-0.595	6.54E-05	-0.608	7.70E-03
10231	*RCAN2*	regulator of calcineurin 2	-1.318	2.79E-03	-1.064	4.78E-03
10446	*LRRN2*	leucine rich repeat neuronal 2	-1.447	5.20E-10	-2.010	3.24E-03
10687	*PNMA2*	PNMA family member 2	-0.641	3.09E-02	-0.926	1.47E-02
10742	*RAI2*	retinoic acid induced 2	-0.612	7.31E-04	-1.019	1.71E-03
10857	*PGRMC1*	progesterone receptor membrane component 1	-0.929	1.46E-15	-0.731	6.33E-04
10924	*SMPDL3A*	sphingomyelin phosphodiesterase acid like 3A	-2.152	1.36E-06	-1.536	6.21E-03
114780	*PKD1L2*	polycystin 1 like 2 (gene/pseudogene)	-0.817	5.42E-09	-0.709	6.29E-03
116039	*OSR2*	odd-skipped related transciption factor 2	-1.260	8.12E-06	-1.098	1.24E-02

1191	*CLU*	clusterin	-1.005	1.09E-04	-1.140	4.56E-03
1193	*CLIC2*	chloride intracellular channel 2	-1.378	4.08E-13	-0.876	1.66E-02
124935	*SLC43A2*	solute carrier family 43 member 2	-0.796	8.87E-04	-0.627	3.06E-02
125488	*TTC39C*	tetratricopeptide repeat domain 39C	-1.087	2.38E-04	-0.639	1.89E-02
131544	*CRYBG3*	crystallin beta-gamma domain containing 3	-1.280	1.83E-05	-0.724	2.14E-02
133	*ADM*	adrenomedullin	-0.999	9.60E-05	-0.809	1.47E-02
140766	*ADAMTS14*	ADAM metallopeptidase with thrombospondin type 1 motif 14	-0.817	3.91E-05	-0.649	3.69E-03
146556	*C16orf89*	chromosome 16 open reading frame 89	-0.899	1.78E-06	-1.176	3.83E-02
147463	*ANKRD29*	ankyrin repeat domain 29	-0.949	2.12E-03	-0.885	2.37E-02
154091	*SLC2A12*	solute carrier family 2 member 12	-1.287	2.36E-03	-0.705	5.24E-03
1591	*CYP24A1*	cytochrome P450 family 24 subfamily A member 1	-3.151	1.53E-08	-1.717	1.17E-03
160335	*TMTC2*	transmembrane O-mannosyltransferase targeting cadherins 2	-0.859	7.18E-03	-0.696	2.25E-02
1803	*DPP4*	dipeptidyl peptidase 4	-0.818	3.14E-03	-0.954	2.26E-02
2009	*EML1*	EMAP like 1	-0.850	3.23E-03	-1.298	5.26E-03
2070	*EYA4*	EYA transcriptional coactivator and phosphatase 4	-2.559	1.75E-04	-1.145	3.49E-03
2192	*FBLN1*	fibulin 1	-1.948	9.38E-04	-1.690	1.43E-02
2201	*FBN2*	fibrillin 2	-2.136	3.39E-03	-1.143	3.76E-02
220108	*FAM124A*	family with sequence similarity 124 member A	-0.821	1.16E-04	-0.656	2.77E-03
220164	*DOK6*	docking protein 6	-1.012	2.21E-04	-1.161	2.71E-03
221400	*TDRD6*	tudor domain containing 6	-0.851	2.85E-09	-0.919	1.89E-03
2273	*FHL1*	four and a half LIM domains 1	-0.660	3.16E-02	-0.740	9.36E-03
22809	*ATF5*	activating transcription factor 5	-0.760	6.75E-07	-1.806	3.44E-03
23024	*PDZRN3*	PDZ domain containing ring finger 3	-1.010	4.07E-04	-0.773	1.70E-02
23102	*TBC1D2B*	TBC1 domain family member 2B	-0.622	2.33E-06	-0.630	2.94E-02
23175	*LPIN1*	lipin 1	-0.740	1.33E-05	-0.869	5.25E-03

23452	*ANGPTL2*	angiopoietin like 2	-1.287	1.75E-04	-0.966	7.87E-06
253738	*EBF3*	EBF transcription factor 3	-1.607	7.95E-06	-1.344	1.36E-02
25816	*TNFAIP8*	TNF alpha induced protein 8	-1.206	7.66E-06	-1.247	6.99E-03
25840	*METTL7A*	methyltransferase like 7A	-2.137	3.71E-05	-1.654	9.95E-04
25854	*FAM149A*	family with sequence similarity 149 member A	-1.060	5.35E-05	-0.995	3.89E-03
25891	*PAMR1*	peptidase domain containing associated with muscle regeneration 1	-1.531	1.64E-04	-1.167	7.32E-03
26002	*MOXD1*	monooxygenase DBH like 1	-1.577	1.80E-03	-0.750	5.47E-03
26112	*CCDC69*	coiled-coil domain containing 69	-1.984	2.94E-07	-0.746	2.75E-03
261729	*STEAP2*	STEAP2 metalloreductase	-0.833	5.73E-03	-0.620	6.15E-03
274	*BIN1*	bridging integrator 1	-0.732	1.34E-06	-0.825	1.35E-02
284611	*FAM102B*	family with sequence similarity 102 member B	-1.514	6.82E-06	-1.073	2.32E-03
285195	*SLC9A9*	solute carrier family 9 member A9	-1.427	2.64E-04	-0.851	1.86E-02
340419	*RSPO2*	R-spondin 2	-1.297	3.30E-03	-1.110	5.00E-03
3671	*ISLR*	immunoglobulin superfamily containing leucine rich repeat	-1.304	8.27E-04	-1.127	4.07E-02
3751	*KCND2*	potassium voltage-gated channel subfamily D member 2	-1.011	9.88E-03	-0.986	5.26E-03
3775	*KCNK1*	potassium two pore domain channel subfamily K member 1	-0.688	4.17E-04	-0.791	1.05E-02
388135	*C15orf59*	chromosome 15 open reading frame 59	-0.848	7.85E-04	-0.973	3.55E-02
4857	*NOVA1*	NOVA alternative splicing regulator 1	-2.049	2.63E-12	-1.063	1.85E-02
5046	*PCSK6*	proprotein convertase subtilisin/kexin type 6	-1.081	1.83E-02	-0.717	3.88E-03
5087	*PBX1*	PBX homeobox 1	-1.239	3.00E-07	-1.246	1.20E-03
54414	*SIAE*	sialic acid acetylesterase	-0.846	2.62E-05	-0.626	2.22E-02
54518	*APBB1IP*	amyloid beta precursor protein binding family B member 1 interacting protein	-1.239	3.80E-03	-1.926	1.77E-03
54756	*IL17RD*	interleukin 17 receptor D	-0.863	9.03E-07	-1.225	3.00E-02
54842	*MFSD6*	major facilitator superfamily domain containing 6	-0.793	5.47E-04	-0.831	1.47E-02

54899	*PXK*	PX domain containing serine/threonine kinase like	-0.707	2.47E-04	-0.840	5.00E-03
5493	*PPL*	periplakin	-0.729	3.62E-07	-1.458	1.53E-03
55034	*MOCOS*	molybdenum cofactor sulfurase	-0.819	1.81E-04	-1.347	8.78E-04
55130	*ARMC4*	armadillo repeat containing 4	-1.497	6.41E-05	-0.594	2.32E-02
55652	*SLC48A1*	solute carrier family 48 member 1	-0.918	4.04E-06	-0.664	2.28E-03
56944	*OLFML3*	olfactomedin like 3	-0.750	1.63E-03	-0.911	3.22E-03
56977	*STOX2*	storkhead box 2	-0.721	1.92E-02	-0.859	4.69E-02
57161	*PELI2*	pellino E3 ubiquitin protein ligase family member 2	-0.856	1.71E-04	-0.932	1.35E-02
57699	*CPNE5*	copine 5	-0.894	2.27E-06	-1.031	7.79E-05
57718	*PPP4R4*	protein phosphatase 4 regulatory subunit 4	-0.638	2.74E-02	-1.158	1.95E-03
5789	*PTPRD*	protein tyrosine phosphatase receptor type D	-1.252	3.17E-06	-2.590	7.34E-04
59084	*ENPP5*	ectonucleotide pyrophosphatase/phosphodiesterase family member 5	-1.886	1.57E-05	-0.706	8.85E-04
5918	*RARRES1*	retinoic acid receptor responder 1	-0.968	8.30E-03	-0.618	3.05E-02
5950	*RBP4*	retinol binding protein 4	-1.917	1.06E-08	-0.686	2.02E-03
5980	*REV3L*	REV3 like, DNA directed polymerase zeta catalytic subunit	-1.121	9.15E-07	-1.150	2.38E-04
604	*BCL6*	BCL6 transcription repressor	-0.672	2.27E-05	-0.693	5.14E-03
6322	*SCML1*	Scm polycomb group protein like 1	-0.736	5.16E-03	-0.739	1.05E-03
64094	*SMOC2*	SPARC related modular calcium binding 2	-1.973	5.13E-03	-3.415	3.86E-05
6414	*SELENOP*	selenoprotein P	-3.846	3.29E-05	-3.075	1.76E-03
642976	*GRIK1-AS1*	GRIK1 antisense RNA 1	-0.945	1.59E-10	-1.005	5.82E-03
64388	*GREM2*	gremlin 2, DAN family BMP antagonist	-2.481	5.40E-06	-2.886	1.92E-04
66004	*LYNX1*	Ly6/neurotoxin 1	-0.681	3.01E-05	-1.074	8.67E-04
7049	*TGFBR3*	transforming growth factor beta receptor 3	-2.228	2.86E-08	-0.704	4.87E-02
7079	*TIMP4*	TIMP metallopeptidase inhibitor 4	-1.169	8.89E-12	-0.681	1.02E-02

7088	*TLE1*	TLE family member 1, transcriptional corepressor	-0.914	1.55E-04	-0.694	2.78E-02
7089	*TLE2*	TLE family member 2, transcriptional corepressor	-0.612	1.79E-04	-0.732	2.05E-02
7373	*COL14A1*	collagen type XIV alpha 1 chain	-2.019	9.93E-04	-1.970	1.42E-02
7436	*VLDLR*	very low density lipoprotein receptor	-1.051	2.68E-05	-1.454	3.26E-03
79789	*CLMN*	calmin	-1.599	1.21E-07	-1.195	3.58E-03
79827	*CLMP*	CXADR like membrane protein	-0.827	7.13E-03	-0.746	1.15E-03
79895	*ATP8B4*	ATPase phospholipid transporting 8B4 (putative)	-1.846	2.00E-07	-1.463	1.16E-04
79961	*DENND2D*	DENN domain containing 2D	-0.862	3.02E-07	-1.247	1.79E-04
80332	*ADAM33*	ADAM metallopeptidase domain 33	-0.764	1.66E-06	-1.114	2.74E-03
84034	*EMILIN2*	elastin microfibril interfacer 2	-0.945	2.11E-04	-1.652	9.64E-03
8406	*SRPX*	sushi repeat containing protein X-linked	-0.979	1.36E-05	-0.675	2.16E-02
84870	*RSPO3*	R-spondin 3	-1.374	3.33E-02	-2.216	1.45E-03
8804	*CREG1*	cellular repressor of E1A stimulated genes 1	-0.595	2.95E-05	-0.710	1.05E-02
8825	*LIN7A*	lin-7 homolog A, crumbs cell polarity complex component	-1.139	3.57E-03	-0.930	9.66E-03
89927	*BMERB1*	bMERB domain containing 1	-1.148	1.72E-05	-0.739	8.50E-03
90139	*TSPAN18*	tetraspanin 18	-1.684	1.88E-04	-1.075	1.17E-02
9096	*TBX18*	T-box transcription factor 18	-0.871	6.72E-05	-0.630	2.13E-03
91851	*CHRDL1*	chordin like 1	-2.989	3.63E-04	-1.593	1.44E-02
93099	*DMKN*	dermokine	-0.865	7.30E-09	-1.449	2.73E-02
9481	*SLC25A27*	solute carrier family 25 member 27	-1.753	1.66E-05	-1.020	1.51E-02
9764	*KIAA0513*	KIAA0513	-0.789	2.58E-05	-0.619	2.92E-02
9770	*RASSF2*	Ras association domain family member 2	-1.713	4.39E-03	-1.578	1.12E-04
9839	*ZEB2*	zinc finger E-box binding homeobox 2	-0.656	6.24E-03	-0.642	2.31E-02
